# Preference for Competitive Employment in People with Mental Disorders: A Systematic Review and Meta-analysis of Proportions

**DOI:** 10.1007/s10926-024-10192-0

**Published:** 2024-04-25

**Authors:** Christine Adamus, Dirk Richter, Kim Sutor, Simeon Joel Zürcher, Sonja Mötteli

**Affiliations:** 1 Centre for Psychiatric Rehabilitation, Universitäre Psychiatrische Dienste Bern (UPD), Bern, Switzerland; 2 University Hospital of Psychiatry and Psychotherapy, University of Bern, Bern, Switzerland; 3 Department of Health Professions, Bern University of Applied Sciences, Bern, Switzerland; 4 Department of Sport and Health Sciences, Technical University of Munich, Munich, Germany; 5 Centre for Psychiatric Rehabilitation, Universitäre Psychiatrische Dienste Bern (UPD), 3098 Köniz, Switzerland

**Keywords:** Preference, Competitive employment, Social inclusion, Mental disorders, Meta-analysis, Proportions

## Abstract

**Purpose:**

The inclusion of people with mental disorders (MD) into competitive employment has become an important political and therapeutic goal. The present paper investigates meta-analytically to which extent people with MD who were unemployed or on sick leave due to MD prefer to work in a competitive job environment.

**Methods:**

For this systematic review and meta-analysis of proportions, we searched Medline, PsycInfo, Cinahl, Google Scholar, and reference lists for peer-reviewed publications from 1990 to Dec 2023, which provided data on the job preferences of people with MD. Two authors independently conducted full-text screening and quality assessments. Pooled proportions of job preferences were calculated with a random-effects meta-analysis of single proportions, and subgroup analyses were performed to examine characteristics associated with job preferences.

**Results:**

We included 30 studies with a total of 11,029 participants in the meta-analysis. The overall proportion of participants who expressed a preference for competitive employment was 0.61 (95%-CI: 0.53–0.68; *I*^*2*^ = 99%). The subgroup analyses showed different preference proportions between world regions where the studies were conducted (*p* < 0.01), publication years (*p* = 0.03), and support settings (*p* = 0.03).

**Conclusion:**

Most people with MD want to work competitively. More efforts should be given to preventive approaches such as support for job retention. Interventions should be initiated at the beginning of the psychiatric treatment when the motivation to work is still high, and barriers are lower.

**Trail Registration:**

The protocol is published in the Open Science registry at https://osf.io/7dj9r

**Supplementary Information:**

The online version contains supplementary material available at 10.1007/s10926-024-10192-0.

## Introduction

Mental disorders (MD) are one of the leading causes of missed educational opportunities, lower educational achievements, sick leave, job loss, long-term unemployment, and social exclusion [1]. However, apart from workplace characteristics such as high demands and low control, which can lead to mental health problems, employment is associated with better health [2]. Unemployment can cause mental distress through loss of structure, social contacts, economic status, activity and other important functions, leading to social exclusion and financial deprivation [3]. For many people with MD, even for those with more prolonged MD or severe mental illness (SMI), employment is an important goal in their recovery process [4–6]. Therefore, supporting a return to work is a core priority of mental health care services [7].

People with prolonged MD perceive several barriers to paid employment, including stigma, lack of skills and confidence, and cognitive and motivational problems caused by psychiatric symptoms and the side effects of pharmaceutical treatments [8]. Several vocational rehabilitation services have been established to support people with MD, including SMI, to return to work. Traditional services train individuals in sheltered pre-vocational training or transitional jobs to enable them to work in the general labour market (first train, then place approach). In contrast, Supported Employment (SE), and particularly Individual Placement and Support (IPS), aim to place individuals directly into the general labour market (first place, then train approach), taking the individual’s preferences and needs into account. IPS is more than twice as effective as traditional vocational approaches [9–11]. Furthermore, its effectiveness implies that competitive employment is possible even for people with SMI. However, employment rates for people with SMI remain low and are estimated to be less than 30% [1, 12].

People with any form of MD have the same rights to make work-related decisions as all other people do [13]. However, this principle is often not put into practice. It is widely assumed that most people with MD want to work competitively [14, 15]. Nevertheless, preference rates for employment in the general labour market of people with MD still need to be systematically reviewed. This systematic review and meta-analysis aimed to estimate the pooled proportion of people who are unemployed or on sick leave due to MD who prefer to work in the general labour market. Knowledge of preference rates for competitive employment enables policymakers and healthcare providers to set realistic goals and priorities to promote the rights of people with MD to work and live an inclusive life.

## Methods

We conducted a systematic review and meta-analysis of peer-reviewed publications reporting preference rates of individuals with MD for competitive employment. We synthesised existing evidence on this topic and assessed contextual factors that may explain differences in preference estimates.

The protocol was published on Dec 5, 2021 (https://osf.io/7dj9r), and the study is reported in adherence to the PRISMA guidelines [16].

### Search Strategy and Selection Criteria

We ran systematic searches on Medline, PsycInfo (both via Ovid) and Cinahl (via EBSCOhost) for peer-reviewed publications from 1990 to Dec 2023. We searched for keywords related to individuals with MD, their preferences, and work (see Supplementary material, Table S1-S2, for the complete search syntax). Additional searches were conducted on Google Scholar and in reference lists of relevant reviews and studies.

Inclusion criteria were peer-reviewed articles of empirical studies providing prevalence data on preferences for competitive employment of individuals with MD aged between 16 and 65 who were unemployed or on sick leave due to MD. Studies published since 1990 and written in Latin letters were included. Studies that did not provide prevalence data on preferences for competitive employment, qualitative studies, and studies that only reported on populations with disabilities other than MD (e.g. mobility, visual, or intellectual disorders) were excluded. Articles not in English or German were translated using DeepL.com to assess their eligibility.

After removing duplicates, two authors (ChA, LE) independently screened the articles based on titles and abstracts, and full-texts were retrieved for closer inspection. Each full-text was independently assessed for eligibility by two authors and blinded to each other’s decisions (ChA, SM). Discrepancies were resolved through discussion involving a third reviewer (DR). If multiple publications were based on the same data, only the first publication was considered in each case.

### Data Extraction and Coding

Two authors independently extracted data for each of the included studies (ChA, SM, KS) using a standardised form. Variables extracted for study description were first author, publication year, country, year of study conduction, study design, sampling method, response rate, support setting (vocational rehabilitation, community mental health care setting, inpatient and outpatient psychiatric treatment setting, other settings), gender ratio, age, type and severity of MD, employment status, education, assessment method for job preferences, total sample size, and target sample size.

The outcome of interest was the number of individuals with a preference for competitive employment (including preferences for job training, education, or Supported Employment services) among the target sample. Competitive employment was defined as any full-time or part-time (self-) employment that paid at least the minimum wage or other usual compensation, with or without professional support (including preferences for education, training, or university studies). Non-competitive employment was defined as any employment situation other than competitive employment and included transitional or sheltered employment, employment without pay, or work in day centres*.* The target sample includes all individuals in the total sample with MD who were unemployed or on sick leave due to MD (e.g. psychiatric inpatients). As recommended in the methodological literature [17], the target sample only included complete cases; subjects with missing answers about job preference were excluded. Because several studies considered different study groups (i.e. subsamples of people with physical impairments or MD), participant characteristics were extracted only when it referred to the subgroup with a majority (> 80%) affected by MD. If a publication only reported on percentages, frequency counts were calculated by the authors. If the preference for competitive employment was reported on a continuum instead of a single value (e.g. strong, moderate, low, no job preference), we extracted the number of individuals with a strong preference. If preferences were reported for different time points (e.g. now, in the near or distant future), we extracted the rate for job preferences in the future.

### Study Risk of Bias Assessment

The quality of the studies was independently assessed by two authors (ChA, SM, KS) using seven of the nine items of the Joanna Briggs Institute (JBI) Critical Appraisal Checklist for Studies Reporting Prevalence Data [18] (Supplementary material, Table S3). Each item (sampling frame, recruitment method, sample size, description of subjects and settings, valid assessment, statistical analysis, and response rate) was rated with yes (1), no (0), or unclear (0), and quality sum scores were computed. A quality sum score of six to seven was classified as good, four to five as moderate, and three or less as poor study quality. The interrater agreement of the quality ratings was 83%. Discrepancies were resolved through discussion. We did not perform publication bias tests because their utility in studies reporting proportions is not clear [19].

### Data Analysis

We calculated the proportion of individuals with MD who preferred competitive employment for each study. A random-effect analysis of single proportions was performed using the inverse variance method to pool the point estimates of job preferences [20]. The Freeman-Tukey double-arcsine transformation was used while pooling the estimates [21]. Results are reported as forest plots showing the pooled proportions and associated 95% confidence intervals (95%-CI). Heterogeneity between the studies was assessed using *I*^2^ and prediction intervals.

Subgroup analyses were performed to explore potential moderating factors that might explain the heterogeneity between proportions across studies. Subgroups were defined during the data extraction process by consensus discussion (ChA, SM, DR), considering knowledge from relevant research. Subgroup analyses were conducted in terms of study quality ratings (high, medium, low), support setting (vocational rehabilitation services, community mental health and other settings, psychiatric treatment settings), the proportion of schizophrenic spectrum disorders in the sample (less than 50%, more than 50%), assessment of job preferences (closed-ended questions asking for preferences to work competitively or to use Supported Employment services, open-ended or multiple choice questions asking for preferences for multiple employment options), study year (before and after the financial crisis in 2008), and world regions of studies (America, Europe, Australia, and Asia). Differences between subgroups were tested using Chi^2^ tests with *α* = 0.05.

By JBI recommendations [22], we did not exclude low-quality studies from the meta-analysis. Instead, we performed sensitivity analyses 1) by excluding the low-quality studies to explore their contribution to the results of the meta-analysis and subgroup comparisons and 2) by excluding studies with inadequate recruitment methods (JBI Q2).

All statistical analyses were conducted using *meta* (version 6.2-1) [23] of the R statistical software (version 4.2.2) [24].

## Results

**Fig. 1:**
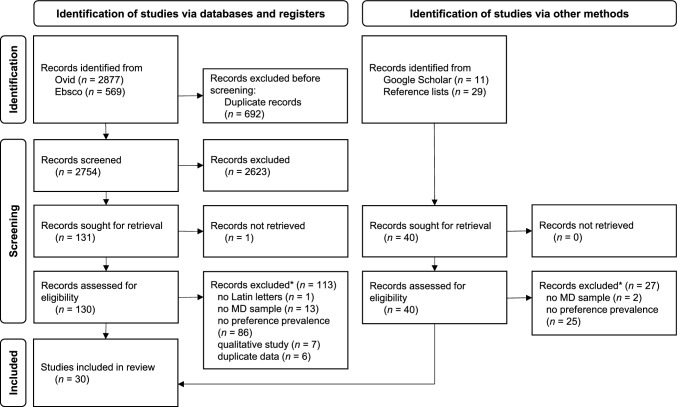
PRISMA flow diagram of study selection (MD = Mental disorder. *Studies may be excluded for multiple reasons; the numbers listed refer to the primary reason for exclusion)

After removing duplicates, we screened the titles and abstracts of 2754 unique database records for eligibility (Fig. 1). We reviewed 131 full-text articles from the database search and 40 from the searches in the reference lists and Google Scholar. Of these, 30 studies were identified as eligible and were included in the systematic review and meta-analysis [14, 25–53].

The studies included 16,062 individuals with sample sizes ranging from 35 to 3380 for single studies (Table 1). The size of the target samples ranged from 16 to 2163 individuals, summing up to a total of 11,029 participants included in the meta-analysis.

**Table 1:** Characteristics of the included studies providing prevalence data on preferences for competitive employment of adult individuals with mental disorders

Study	Country	Design	Sex (% f)	Age (mean)	Support setting	Mental disorders	Empl (gen LM)	Empl (other)	Total samp size	Target samp size	Assessment	Single prop	JBI quality
Ali et al. [25]	USA	Retrospective cross-sectional	63.8	44.8	General population	Emotional or mental disability	39.7	NA	2273	99^a^	Single survey item: "Would you like to have a paid job, either now or in the future?" (yes // no)	89.9	Medium (5/7)
Bonsaksen et al. [26]	Norway	Prospective cross-sectional	48.3	NA	Community mental health care	History of mental illness	14.0	48.1	87	87	Single survey item: "Do you have a desire to get into work?” (yes // maybe, no)	55.2	Medium (5/7)
Briest [27]	Germany	Prospective cross-sectional	72.2	51.4	General population	Mental and behavioural disorders (ICD-10; F00–F99); receiving a temporary disability pension due to mental illness	0.0	9.4	3380	2163^a^	Short-Form-36 Health Survey (SF-36): "General desire to return to employment" (rating scale 0 (not at all) to 10 (in any case); cut-off ≥ 7)	27.8	High (6/7)
Camardese and Youngman [28]	USA	Prospective cross-sectional	34.0	NA	Community mental health care	Homeless individuals with severe and persistent mental illness	0.0	NA	100	100	Open interview questions on vocational aspirations and support needs (expressed a desire for working)	44.0	Low (3/7)
Casper and Carloni [29]	USA	Prospective cross-sectional	46.0	46.8	Community mental health care	Serious mental illness (DSM-IV Axis II diagnosis, duration, and disability)	21.0	NA	345^a^	269	Consumers’ Employment Services Referral Decision (CERSD): Question about whether participants would accept a referral to Supported Employment services (within the next 6 months // not at all in the next 6 months)	49.1	High (6/7)
Drebing et al. [30]	USA	Retrospective cross-sectional	1.3	45.5	Vocational rehabilitation	Veterans with severe mental illness: Affective disorder 32.5%, PTSD 23.2%, Anxiety 19.3%, Bipolar 12.3%, Schizophrenia 3.9%, Other psychosis 2.2%; Any psychiatric disorder 55.4%; Comorbid psychiatric and substance use disorders 46.4%; Alcohol abuse or dependence 47.4%, Drug abuse or dependence 73.2%, Any substance abuse or dependence 82.0%; No psychiatric or substance abuse, medical problem only 8.8%	NA	NA	228	228	Single interview item: “What do you hope to gain from participation in Compensated Work Therapy?” (competitive job was identified as a relevant goal on a list with 13 possible goals)	52.6	Medium (4/7)
Eikelmann and Reker [31]	Germany	Prospective cross-sectional	37.6	36.0	Vocational rehabilitation	Mental and behavioral disorders (ICD-10): F2 60.2%, F4 10.8%, F7 11%, F3 7%, F0 6.8%, F1 4.7%, F8 0.4%, Unclarified diagnosis 1.2%; Living in dependent residential system 78%	0.0	100.0	502	502	Single interview item asking for expectations for the next 12 months (change to the open labour market // change to or remain in any sheltered labour market, change to unemployment, unclear)	17.7	High (6/7)
Filia et al. [32]	Australia	Prospective cross-sectional	37.5	39.6	Psychiatric: Inpatient & Outpatient	Bipolar Disorder (DSM-IV-TR)	54.3	NA	35	16^a^	NA (indicated that they currently wished to be employed)	87.5	Low (3/7)
Frounfelker at al. [33]	USA	Retrospective cross-sectional	38.6	39.1	Community mental health care	Mental and behavioral disorders (DSM-IV): Bipolar disorder 28.5%, Schizophrenia 18.1%, Schizoaffective 13.6%, Major depression 21.1%, Depressive disorder not otherwise specified 7.1%, Psychotic disorder not otherwise specified 4.98%, Other 6.5%, Co-occurring substance disorder 34%; History of homelessness 15.3%	6.1	NA	1748	1748	Single item asking for interest in Supported Employment services (yes // no)	71.8	High (7/7)
Graffam and Naccarella [34]	Australia	Prospective cross-sectional	18.7	NA	Vocational rehabilitation	Schizophrenia 37.43%, Affective Disorder 13.2%, Depression 9.9%, Schizo-Affective Disorder 8.8%, Bipolar Disorder 7.7%, Personality Disorder 3.3%, Dual-Diagnosis 2.2%, Drug-Induced Psychosis 1.1%	0.0	0.0	91	91	Single questionnaire item asking for the motivation to work (very keen // somewhat keen, not keen)	68.1	Low (3/7)
Gühne et al. [35]	Germany	Prospective cross-sectional	56.1	42.7	Psychiatric: Inpatient and Outpatient	Serious mental illness (ICD-10 diagnosis, duration, and disability): F2x 30.8%; F32, F33 59.3%; F30, F31 9.9%	27.9	23.0	383	383	Attitudes and Knowledge Regarding Psychosocial Therapies questionnaire item: “How strong is your current desire for paid employment in the general labour market?” (strong // medium, low)	59.8	High (6/7)
Hatfield [36]	GB	Prospective cross-sectional	45.0	NA	Community mental health care (rehabilitation) and psychiatric treatment (inpatient and outpatient)	Severe and long-term mental illness: available diagnosis 72.5%, schizophrenia 56.7%	8.3	20.8	120^a^	59	Social Interview Schedule (SIS) item: "Would you like help in getting back a job?" (yes // no)	49.2	Medium (4/7)
Henry et al. [37]	USA	Prospective cross-sectional	NA	NA	General population	Mental or emotional problems (self-reported)	44.0	0.0	1093	374	The Mass Health Employment and Disability Survey (MHEDS) item asks for future work intentions (currently looking or planning to look for work in the next few years // neither looking nor planning to look for work)	38.8	Medium (5/7)
Hölzle et al. [38]	Germany	Intervention study	58.5	43.1	Psychiatric: Inpatient and Outpatient	Mental disorders: F2 19.5%, F3 64.6%, F4 15.9%	100.0	NA	82	82	Single interview item asking if a return to work is desired (desired // unclear, not desired)	76.8	Medium (5/7)
Iyer et al. [39]	India	Prospective cross-sectional	55.9	28.7	Psychiatric treatment: Outpatient	Schizophrenia spectrum disorder (DSM-IV): Schizophrenia 58.8%, Schizoaffective 22.1%, Psychotic disorder not otherwise specified 19.1%	NA	NA	68	68	Goal Attainment section of the Wisconsin Quality of Life – Client Questionnaire: "What do you hope to accomplish as a result of your mental health treatment? Please write below up to three goals" (Identification of an employment-related goal as a primary, second or third goal: thematic goal category: work or school)	76.5	Medium (4/7)
Khare et al. [40]	India	Prospective cross-sectional	69.8	40.1	Psychiatric treatment: Outpatient	Mental disorders: Schizophrenia spectrum disorder 69.3%, Major mood disorder 30.7%	60.9	0.0	542	212^a^	Single interview item asking participants whether they would be interested in working, either currently or in the future (yes // no)	77.4	Medium (4/7)
Khare et al. [41]	India	Prospective cross-sectional	34.7	39.3	Psychiatric treatment: Outpatient	Serious mental illness (medical records): Schizophrenia-schizoaffective disorder 90%, Major mood disorder 10%	40.0	0.0	150^a^	90	Single interview item asking participants whether they would be interested in working, either currently or in the future (yes // no)	92.2	High (6/7)
Knaeps et al. [42]	Belgium	Prospective cross-sectional	50.7	42.0	Psychiatric hospital: Inpatient (93%), Community mental health care (rehabilitation) (7%)	Mental health problems: Mood and anxiety 40.9%, Substance-related 28.5%, Psychotic 23.5%, Personality 30.2%, Multiple cognitive 93 (13.1%), Other (e.g. ABI, cognitive...) 7.1%; Multiple mental health problems (clinicians' declaration) 38.7%	22.6	NA	733	733	Multiple response questionnaire questions to indicate long-term vocational goals (competitive employment, self-employment, education // no activity, sheltered employment, voluntary work, day activity centre, domestic work, other, and ‘‘work experience program’’)	58.3	High (6/7)
Laudet et al. [43]	USA	Prospective longitudinal	31.0	40.0	Self-help program	Dually diagnosed persons; Primary diagnosis of mental illness (self-reported): Schizophrenia 40%, Unipolar (major) depression 21%, Bipolar disorder 21%, Schizoaffective 8%, Mood disorder 4%, Other 6%; Primary substance (lifetime abuse): Crack/cocaine 41%, Alcohol 35%, Heroin 11%, Marijuana 10%, Other 3%, Any substance use past year 30%	0.0	0.0	130	130	Open-ended interview item: “What do you hope to do, change, accomplish in the next year?” (mentioned getting a job, or training or gaining skills to get a job, as a goal)	47.7	Medium (4/7)
Macias et al. [44]	USA	Intervention study	45.0	39.0	Vocational rehabilitation	Serious mental illness (DSM-IV): Schizophrenia spectrum disorder 52%, history of serious substance abuse 61%	0.0	0.0	166	166	Single interview item: "Are you currently interested in competitive working / paid employment?" (yes // uncertain, no)	70.5	Medium (4/7)
McQuilken et al. [45]	USA	Prospective cross-sectional	45.0	40.7	Community mental health care and outpatient treatment	Severe mental illness (agency psychiatrists): Schizophrenia 35%, Schizoaffective disorder 25%, Bipolar disorder 20% and Major depression 12%	16.0	NA	369^a^	310	Single interview item asking for participants' desire to work (want to work and were actively looking for work, want to work but were not looking // want not to work)	54.8	Medium (5/7)
Mueser et al. [46]	USA	Intervention study	33.9	29.6	Psychiatric: Inpatient and Outpatient	Schizophrenia spectrum disorder (SCID for DSM-III-R): Schizophrenia 79.6%, Schizoaffective disorder 13.1%, Schizophreniform disorder 7.3%	9.7	6.8	313^a^	233	Desire for / Interest in work (yes // no)	58.8	Low (3/7)
Poremski et al. [47]	Canada	Intervention study	32.7	40.9	Community mental health care	Homeless individuals with mental disorders (MINI, DSM-IV): Psychotic disorder 44.0%, Major depressive disorder 33.6%, Mania or hypomania 13.6%, Mood disorder with psychotic features 4.3%, Posttraumatic stress disorder 2.5%, Panic disorder 2.0%	4.1	NA	2085^a^	2000	NA (expressed a desire for competitive/paid employment in the community)	77.0	Low (3/7)
Ramsay et al. [48]	USA	Prospective cross-sectional	26.0	24.3	Psychiatric hospital	First episode of nonaffective psychotic disorder (SCID, DSM-IV): Schizophrenia 56.0%, Psychotic disorder not otherwise specified 17.0%, Schizoaffective disorder 14.0%, Schizophreniform disorder 8.0%, Delusional disorder 3.0%, Brief psychotic disorder 2.0%; Alcohol use disorder (SCID): Abuse 12.9%, Dependence 20.4%; Cannabis use disorder: Abuse 15.2%, Dependence 44.6%	33.0	NA	100	100	Single interview item: "If services were available, would you like assistance from mental health professionals with [finding a job]?" (yes // no)	80.0	Medium (4/7)
Rennhack et al. [49]	Switzerland	Intervention study	42.9	37.4	Vocational rehabilitation for inpatient and day hospital clients	Mental and behavioral disorders (ICD-10): F1 19.4%, F2 18.4%, F3 34.7%, F4 16.3%, F6 8.1%, Other diagnosis groups 3.1%	28.6	11.2	98	98	Single item asking for the desired productivity status at admission to pre-vocational therapy (employment on the regular job market, education or training on the regular job market // vocational integration programme, employment on the protected job market, education or training on the protected job market, unpaid work or other productive activity, actively looking for a job on the regular job market, actively looking for a job on the protected job market, and no productive activity)	40.8	Medium (4/7)
Secker et al. [50]	GB	Prospective cross-sectional	NA	NA	Community mental health care	People with mental health problems on Care Programme Approach (CPA) levels two and three	4.5	46.2	156	149	Selection of one long-term goal from a given list of opportunities (full-time employment, education, training, self-employment, part-time employment // voluntary work, sheltered work, work experience, job preparation courses, other, don't know)	59.7	Medium (5/7)
Secker and Gelling [51]	GB	Prospective cross-sectional	52.0	NA	Community mental health care	People with mental health problems on enhanced Care Programme Approach (CPA)	13.7	30.3	241^a^	193	Single interview item asking participants if they would be interested in obtaining paid work (now/yes, in the future/maybe // no/not)	71.0	Medium (5/7)
Serowik et al. [52]	USA	Mixed methods study	55.0	46.1	Psychiatric hospital: Inpatient	People with mental disorders (chart review) who were either homeless or had a psychiatric hospitalization in the past 3 months: Bipolar Disorder 42.9%, Schizophrenia 38.8%, Major Depression 34.7%, Personality Disorder 34.7%, Posttraumatic Stress Disorder 8.2%, Anxiety Disorder 6.1%	0.0	NA	49	49	Open interview questions focussing on participants' current and past experience with money and what, if anything, they would like to see changed with regard to their finances (spontaneously expressed a desire to work in relation to their money management)	40.8	Medium (4/7)
Westcott et al. [14]	Australia	Prospective cross-sectional	32.2	NA	Community mental health care	Schizophrenia spectrum disorder (DSM-IV): Schizophrenia 94.1%, Schizoaffective disorder 5.8%	34.5	20.0	255^a^	167	Socially Valued Role Classification Scale (SRCS): "Are you interested in employment as a goal for the future?" (yes/no) and "If so, can you describe what kind of work you would be interested in doing?" (employment, education and training // rehabilitation, caring for others home duties and self-care))	79.0	Medium (4/7)
Zaniboni et al. [53]	Italy	Prospective cross-sectional	36.2	41.0	Vocational rehabilitation	Mental disorders (self-reported): Schizophrenia 25%, other psychotic disorders 19%, depression 12.1%, personality disorder 9.5%, anxiety disorder 3.4%, other psychiatric disorders, e.g. substance abuse 31%	0.0	100.0	140^a^	130	Working Plans (WP) rating scale asking on a 5-point scale for the intentions to a) work in a public or private organization in the regular labour market, b) undertake freelance work, c) keep working at Social Enterprises, d) stop working (Cluster 1 of hierarchical cluster analysis of answers indicating strong intentions to work in a competitive labour market)	30.0	Medium (4/7)

^a^Numerical description of sex, age, mental disorders, and employment refers to this sample (total sample size or target sample size);

// indicates the cut-off for categorical response scales (e.g. preference for competitive employment // no preference for competitive employment)

f = females; Empl. = Employment; gen = general; LM = labour market; samp = sample; prop = proportion; JBI = Joanna Briggs Institute

Twelve studies were conducted in the United States, four in Germany, three in the United Kingdom, Australia, and India, two in Belgium, and one in Italy, Norway, and Switzerland. Studies were published between 1992 and 2021, with 13 studies published before 2008 (1992 to 2007) and 17 published after 2008 (2011 to 2021).

The studies included between 1.3% and 72.2% female participants, and mean age ranged from 24.3 to 51.4 years. Several clinical and social sample characteristics were not or only incompletely reported. For example, the reported MD varied from “history of mental illness” [26] over “mental or emotional problems” [37] and “homeless individuals with severe and persistent mental illness “ [28] to the number or proportion of specific diagnoses in the sample. Of the studies that reported diagnostic information, twelve included fewer than 50% with a schizophrenia spectrum disorder, and ten included more than 50%.

Six studies were conducted in a vocational rehabilitation setting, ten in community mental health settings, four studies were conducted in “other” settings (normal population, self-help programmes), and ten studies were conducted in inpatient and outpatient psychiatric treatment settings. Two vocational settings aimed to reintegrate their service users into competitive employment (Supported Employment settings) [34, 44], and four targeted unspecific or sheltered employment [30, 31, 49, 53].

Job preferences were assessed using a variety of methods. Most studies (*n* = 15) used a single closed-ended question asking participants whether they wished to work in a competitive job or asking them about the intensity of their job preference. Some asked for job preferences within a particular time frame, while most asked about job preferences without a time reference. Three studies asked about the wish to use a Supported Employment service to attain a regular job. Other studies asked for vocational preferences using open-ended questions (*n* = 5) or a list of multiple vocational options (multiple choice; *n* = 5) [14, 42, 49, 50, 53]. Open-ended questions asked participants about their vocational aspirations [28], goals relevant to their participation in the vocational rehabilitation support programme [30], what they hope to accomplish as a result of their mental health treatment [39], what they hope to do, change, or accomplish in the next year [43], and what they would like to see changed with regard to their finances [52]. Multiple-choice questions asked participants to identify preferred vocational goals out of a list with multiple vocational options like competitive employment, self-employment, education and training, freelance work, sheltered employment or vocational rehabilitation, day activity centres, voluntary work, domestic work, and no vocational activity. Two studies did not describe the assessment method [32, 47]. These studies were categorised into the first assessment subgroup (closed-ended questions) based on their description of the findings.

Study quality ratings ranged from 3 to 7 out of 7 possible scores (Table 1 and Supplementary material, Table S4). Our assessment classified the quality of seven studies as high, 18 as medium, and five as low. Overall, study quality was low regarding recruitment procedure and sample size (Supplementary material, Table S4). Study quality was high regarding the sampling frame, description of subjects and settings, assessment methods, and statistical analysis.

**Fig. 2:**
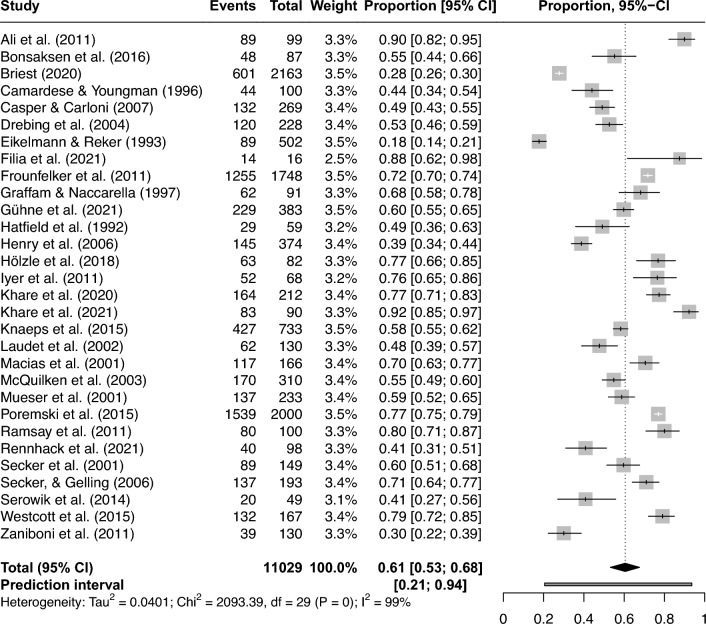
Forest plot of pooled proportions of people with mental disorders who prefer competitive employment

Single preference rates in the individual studies ranged between 17.7 and 92.2%. The meta-analysis revealed a pooled proportion of 0.61 individuals who prefer competitive employment (95%-CI 0.53 to 0.68; Fig. 2). Study heterogeneity was substantial; the overall *I*^*2*^ statistic for heterogeneity was 99%, and the prediction interval ranged from 0.21 to 0.94.

**Fig. 3:**
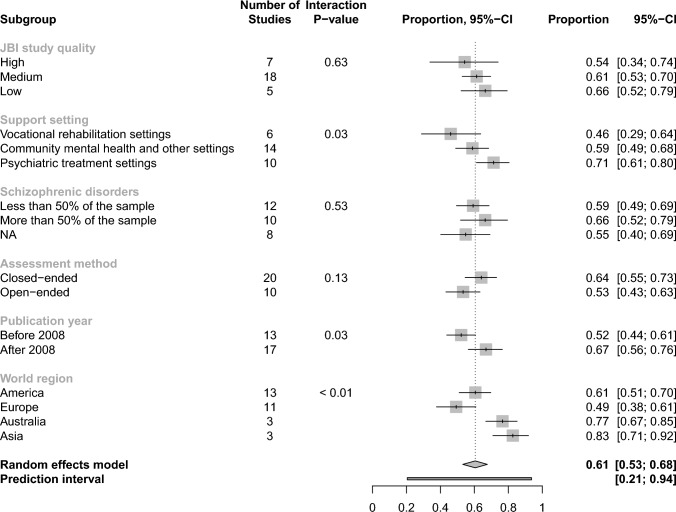
Subgroup analyses of pooled proportions of people with mental disorders who prefer competitive employment (The forest plots of subgroup analyses showing the individual studies and their subgroup assignments are shown in the Supplementary material, Fig. S1-S6. JBI = Joanna Briggs Institute, NA = not available)

Figure 3 shows the subgroup comparisons. Details are presented in Supplementary material, Fig. S1-S6. The subgroup analyses comparing study quality ratings, the proportion of people with schizophrenic disorders, and the assessment methods revealed no significant differences. Subgroups significantly differed regarding the support settings, publication years, and the world regions where the studies had been conducted.

Preference proportions from studies that were conducted in Australia and Asia were higher (0.77; 95%-CI 0.67 to 0.85; *I*^*2*^ = 57% and 0.83; 95%-CI 0.71 to 0.92; *I*^*2*^ = 83% respectively) than those conducted in America (0.61; 95%-CI 0.51 to 0.70; *I*^*2*^ = 97%) or Europe (0.49; 95%-CI 0.38 to 0.61; *I*^*2*^ = 98%). Regarding support settings, preference proportions were highest in psychiatric treatment settings (0.71; 95%-CI 0.61 to 0.80; *I*^*2*^ = 92%), followed by community mental health and other settings (0.59; 95%-CI 0.49 to 0.68; *I*^*2*^ = 99%). Vocational rehabilitation settings showed the lowest preference proportion (0.46; 95%-CI 0.29 to 0.64; *I*^*2*^ = 98%). Among the vocational rehabilitation settings, the Supported Employment settings targeting competitive employment [34, 44] show preference proportions higher than the overall proportion, while the vocational rehabilitation settings targeting unspecific or sheltered employment [30, 31, 49, 53] show preference proportions lower than the overall preference proportion (Supplementary material, Fig. S2). Studies published before 2008 reported smaller preference proportions (0.52; 95%-CI 0.44 to 0.61; *I*^*2*^ = 97%) than studies published after 2008 (0.67; 95%-CI 0.56 to 0.76; *I*^*2*^ = 99%). Regarding assessment methods, there is a trend (*p* = 0.13) for larger preference proportions if assessed with closed-ended questions asking participants whether they wanted to work or wished access to Supported Employment services (0.64; 95%-CI 0.55 to 0.73; *I*^*2*^ = 99%). Preference proportions were smaller when job preferences were indirectly assessed using open-ended or multiple-choice questions (0.53; 95%-CI 0.43 to 0.63; *I*^*2*^ = 92%).

The sensitivity analyses (Supplementary material, Fig. S7-S8) showed no difference in the pooled proportion of job preferences after excluding low-quality studies (0.59; 95%-CI 0.51 to 0.67; *I*^*2*^ = 99%; *k* = 25) or studies with inadequate recruitment methods (0.61; 95%-CI 0.42 to 0.78; *I*^*2*^ = 100%; *k* = 9).

## Discussion

We conducted a systematic review and meta-analysis of proportions among 30 studies that asked individuals with MD who were unemployed or on sick leave due to MD about their preference for competitive employment. This is the first study that systematically synthesises reported preference proportions into a meta-analysis. The pooled analysis showed that 61% of study participants prefer to work competitively. The subgroup analyses showed that the preference proportion varies according to the support setting, world region, and publication year. These findings suggest that preferences are not static but dynamic and malleable, influenced by socio-cultural and economic factors.

The differences we found between the world regions suggest that socio-economic and cultural factors may influence individuals' job preferences. Socio-economic factors such as lower economic development, unequal income distributions, or weak unemployment protection further reinforce the adverse effects of unemployment on mental health [3]. Thus, it is conceivable that these factors also increase preferences for competitive employment. In contrast, fear of losing social security benefits was a significant barrier to employment in more developed countries and increased the likelihood of preferring non-employment [30, 45, 50, 51]. This may explain the lower preference proportion that we found in the European and American studies. Good unemployment and social security insurance guarantees during the job resumption process could support people with MD in pursuing their preferences for a competitive job. In terms of cultural factors, it is known that Asian cultures promote specific work ethics, which may explain some of the differences [54].

In our study, the job preference prevalence was higher in inpatient and outpatient psychiatric treatment settings than in vocational rehabilitation services. This may be related to the fact that more people in psychiatric treatment settings are on sick leave, while most people in vocational rehabilitation settings are unemployed. The barriers to maintaining employment and returning to work after sick leave are lower than for reintegration into new employment [55]. In addition, service users’ preferences for competitive employment were in line with the effectiveness of their vocational rehabilitation services. While Supported Employment services consistently showed to be more effective than traditional pre-vocational services [9–11], preferences for competitive employment were apparently higher in the Supported Employment studies [34, 44] than in those studies whose vocational rehabilitation service targeted unspecific or sheltered employment [30, 31, 49, 53]. This also may be related to the long time spent in psychiatric rehabilitation, which seems to make people with MD fear re-employment and resign themselves to their situation [31, 36, 38]. Therefore, vocational support efforts should begin as early as possible in the mental health recovery process, when their motivation to work is still high, and barriers to work are smaller. For example, workplace interventions combined with therapeutic interventions showed good effectiveness for people on sick leave due to MD [56, 57], and Supported Education programmes could be an appropriate intervention to support young people with MD [58].

With the duration of the mental disorder, the risk for social exclusion increases regarding work and other areas of life. Prolonged and frequent psychiatric hospitalisations are significantly associated with social exclusion regarding employment, housing, family situations, and decreased friendship contacts [59]. The more life domains are affected by social exclusion, the more likely work becomes just one priority among many others. This may also be reflected in our study's different preference proportions across assessment methods. Preferences for competitive employment were higher when asked directly with closed-ended questions than when assessed by open-ended or multiple-choice questions. This finding may suggest that people with MD indeed prefer being included in competitive employment. However, if different goals or support needs compete, people with MD must prioritise.

This study has some limitations. The included studies showed considerable heterogeneity in the reported preference proportions, study quality, support settings, mental disorders, and assessment methods. The wide prediction interval in job preference proportions may comprise the interpretation and may limit our findings’ generalisability. Secondly, only few of the included studies were rated as high-methodology papers. In most studies, the quality was rated low regarding recruitment methods and sample sizes, which may have led to biased estimates and low precision. More high-quality research on the job preferences of people with MD is needed to clarify the influence of methodological heterogeneity on the estimated preference proportion. Thirdly, findings from subgroup analyses should be considered exploratory and hypothesis-generating.

The results of this study show that most individuals with MD want to work competitively. However, to date, less than 30% of them are included in the general labour market [1, 12]. Considering the UN Convention on the Rights of People with Disabilities [13], this gap implies the need for more effective vocational support, such as Supported Employment services. Vocational interventions should be offered in different settings and initiated early in mental health treatment and care when the motivation to work is still high, and barriers to re-employment are lower. The greater the barriers to work have become for people with MD (e.g. through delayed work integration assistance after having already lost employment or through longer treatment paths following the first train, then place approach) [9–11, 55, 59], the more their motivation may be downregulated, which may result in subsequent social exclusion. To support people with MD in realising their right to work and social inclusion, we need to incentivise rather than sanction the return to work (e.g. through loss of social security insurance) and focus on job retention besides reintegration into the general labour market.

## Supplementary Information

Below is the link to the electronic supplementary material.

Supplementary file1 (PDF 2615 KB)

## Data Availability

The authors confirm that all data generated or analysed during this study are included in this published article. The R code of the meta-analysis can be accessed upon request at the corresponding author.
