# Supplementary material for: Preference for Competitive Employment in People with Mental Disorders: A Systematic Review and Meta-analysis of Proportions
Source: J Occup Rehabil. 2024 Apr 25;35(2):143–58. doi: 10.1007/s10926-024-10192-0 (PMC12089194; doi:10.1007/s10926-024-10192-0)
Supplement: Supplementary file 1 — Supplementary file1 (PDF 2615 KB) [file 10926_2024_10192_MOESM1_ESM.pdf]

Adamus, Richter, Sutor, Zürcher, & Mötteli. Preference for competitive employment in people with mental disorders – A systematic review and meta-analysis of proportions.

Supplementary Material

Index

Full search strategy 2
JBI Quality Criteria for prevalence studies 3
JBI Quality Ratings 5
Subgroup Analyses 6
Sensitivity Analyses 12
Excluded papers 13

Correspondence to:

Dr Christine Adamus, Centre for Psychiatric Rehabilitation, Universitäre Psychiatrische Dienste Bern (UPD), 3098 Köniz, Switzerland

christine.adamus@unibe.ch

Full search strategy

Table S1 Ovid (Medline, PsycInfo)

Population	((mental or mentally or psychiatr*).ti,ab. AND
Outcome	(prefer* or choice* or attitude* or choose* or desire* or intend* or intent* or wish* or favo?r* or volition*).ti,ab. AND
Condition	(job or employ* or vocation* or (labo?r adj market*) or career* or occup* or work*).ti.)
	Population AND Outcome AND Condition
Exclusion	NOT (burnout* or overwork* or undergraduate* or student* or paramedic* or gerontolog* or p?ediatr* or oncolog* or cancer* or physician* or surg* or implant* or (intensive adj care) or ICU or fMRI or stroke or child* or newborn* or retire* or nurs* or healthcare or workforce or COVID).ti.
Filter	Humans, Publication date 1990 until recent, Abstract available, Peer reviewed journal, Remove duplicates

Table S2 EBSCOhost (Cinahl)

Population	(TI (mental or mentally or psychiatr*) OR AB (mental or mentally or psychiatry*)) AND
Outcome	((TI (prefer* or choice* or attitude* or choose* or desire* or intend* or intent* or wish* or favo?r* or volition*) OR (AB (prefer* or choice* or attitude* or choose* or desire* or intend* or intent* or wish* or favo?r* or volition*)))) AND
Condition	(TI (job or employ* or vocation* or (lab* market) or career* or occup* or work*))
Exclusion	NOT (TI (burnout* or overwork* or undergraduate* or student* or paramedic* or gerontolog* or p?ediatr* or oncolog* or cancer* or physician* or surg* or implant* or (intensive care) or ICU or fMRI or stroke or child* or newborn* or retire* or nurs* or healthcare or workforce or COVID))
Filter	Humans, Publication date 1990 until recent, Abstract available, Peer reviewed journal, Exclude MEDLINE records

JBI Quality Criteria for prevalence studies

Table S3 JBI quality criteria

Variable Name	Full question	YES (1 Point)	NO (0 Point)	Unclear (0 Points)
**Q1: Sampling Frame**	Was the sample frame appropriate to assess the target population?	Appropriate sample frame for our purpose within a clear defined setting: Working-aged adults with MD	No clear or inappropriate sampling frame for our purpose	No information available
**Q2: Recruitment**	Were study participants recruited in an appropriate way?	Random or complete sampling<br>AND<br>Clear report / description of how sampling was performed	No appropriate sampling method (e.g., snowball sampling / convenience sampling, incomplete sampling)	No clear report / description / declaration of how sampling was performed
**Q3: Sample Size**	Was the sample size adequate?	Sample size calculation was done regarding adult MD Sample<br>OR<br>Large adult MD sample size of at least 300	No sample size calculation<br>AND<br>Sample size of adult MD (sub)sample below 300	Sample size not declared / unclear (e.g., studies that calculated prevalence rates just for a subset of individuals, and it is not clear which subset was used for this calculation)
**Q4: Description of Subjects and Setting**	Were the study subjects and setting described in detail?	A minimal description must include the following:<br>Subjects:<br>- sex ratio,<br>- age (any information on age),<br>- employment status,<br>- MD (minimal description of / if)<br>Setting:<br>- Country/ region,<br>- Setting: inpatient/ outpatient/ community / normal population with MD / prison etc.	At least one item of minimal description is missing	When the target sample (adults with MD) builds only a subsample of the study sample, and minimal description was only provided regarding the whole study sample, but not regarding our purposes

**Q6: Valid Assessment**	Were valid methods used for the identification of the condition?	Self-reported preferences (e.g., single-item questions) or validated self-reported scales to assess preferences<br>AND<br>Appropriately defined labour market (e.g., general labour market / competitive employment)	e.g., Observer reported / rated preferences	Assessment methods unclear (e.g., vague definition of preference or labour market)
**Q8: Statistical Analysis**	Was there an appropriate statistical analysis?	The numerator and denominator of reported prevalence are clearly reported (or transparent)	No clear reporting of numerator / denominator (typically: only percentages for prevalence rates provided)	*not applicable*
**Q9: Response Rate**	Was the response rate adequate, and if not, was the low response rate managed appropriately?	Response rate of at least 70% (included divided by eligible and contacted)<br>OR<br>Discussion of non-response rate (reasons, consequences)<br>OR<br>Comparison of responders and non-responders	Response rate < 70%<br>OR<br>No response rate reported (denominator unclear / unable to calculate response rate)<br>AND<br>No discussion regarding the influence of non-response rate on outcomes	*not applicable*

JBI Quality Ratings

Table S4 JBI quality ratings of the included studies

Study	Q1 Sampling Frame	Q2 Recruitment	Q3 Sample Size	Q4 Description	Q6 Assessment	Q8 Analysis	Q9 Response Rate	JBI Score	Quality Rating
Ali et al. (2011)	YES	YES	NO	YES	YES	NO	YES	5/7	Medium
Bonsaksen et al. (2016)	NO	YES	NO	YES	YES	YES	YES	5/7	Medium
Briest (2020)	YES	YES	YES	YES	YES	NO	YES	6/7	High
Camardese & Youngman (1996)	YES	NO	NO	NO	YES	YES	NO	3/7	Low
Casper & Carloni (2007)	YES	YES	NO	YES	YES	YES	YES	6/7	High
Drebing et al. (2004)	YES	NO	NO	YES	YES	NO	YES	4/7	Medium
Eikelmann & Reker (1993)	YES	YES	YES	YES	NO	YES	YES	6/7	High
Filia et al. (2021)	YES	unclear	NO	YES	unclear	YES	NO	3/7	Low
Frounfelker et al. (2011)	YES	YES	YES	YES	YES	YES	YES	7/7	High
Graffam & Naccarella (1997)	YES	NO	NO	YES	YES	NO	NO	3/7	Low
Gühne et al. (2021)	YES	NO	YES	YES	YES	YES	YES	6/7	High
Hatfield et al. (1992)	YES	NO	NO	YES	unclear	YES	YES	4/7	Medium
Henry et al. (2006)	YES	unclear	YES	unclear	YES	YES	YES	5/7	Medium
Hölzle et al. (2018)	YES	NO	NO	YES	YES	YES	YES	5/7	Medium
Iyer et al. (2011)	YES	unclear	NO	NO	YES	YES	YES	4/7	Medium
Khare et al. (2020)	YES	NO	NO	YES	YES	YES	NO	4/7	Medium
Khare et al. (2021)	YES	YES	NO	YES	YES	YES	YES	6/7	High
Knaeps et al. (2015)	YES	unclear	YES	YES	YES	YES	YES	6/7	High
Laudet et al. (2002)	YES	NO	NO	YES	YES	NO	YES	4/7	Medium
Macias et al. (2001)	YES	NO	NO	YES	YES	YES	NO	4/7	Medium
McQuilken et al. (2003)	YES	NO	YES	YES	YES	YES	NO	5/7	Medium
Mueser et al. (2001)	YES	unclear	NO	YES	unclear	YES	NO	3/7	Low
Poremski et al. (2015)	YES	YES	NO	YES	unclear	NO	NO	3/7	Low
Ramsay et al. (2011)	YES	NO	NO	YES	YES	YES	NO	4/7	Medium
Rennhack et al.(2021)	YES	NO	NO	YES	YES	YES	NO	4/7	Medium
Secker et al. (2001)	YES	YES	NO	NO	YES	YES	YES	5/7	Medium
Secker, & Gelling (2006)	YES	unclear	NO	YES	YES	YES	YES	5/7	Medium
Serowik et al. (2014)	YES	NO	NO	YES	YES	YES	NO	4/7	Medium
Westcott et al. (2015)	YES	NO	NO	YES	YES	YES	NO	4/7	Medium
Zaniboni et al. (2011)	YES	NO	NO	YES	YES	YES	NO	4/7	Medium
**Total**	**29**	**9**	**7**	**26**	**25**	**24**	**17**	**137/210**	

Subgroup Analyses

Fig. S1 Subgroup analysis of quality ratings

Study or Subgroup	Events	Total	Weight	Proportion [95% CI]
JBI study quality = High				
Briest (2020)	601	2163	3.5%	0.28 [0.26; 0.30]
Casper & Carloni (2007)	132	269	3.4%	0.49 [0.43; 0.55]
Eikelmann & Reker (1993)	89	502	3.5%	0.18 [0.14; 0.21]
Frounfelker et al. (2011)	1255	1748	3.5%	0.72 [0.70; 0.74]
Gühne et al. (2021)	229	383	3.5%	0.60 [0.55; 0.65]
Khare et al. (2021)	83	90	3.3%	0.92 [0.85; 0.97]
Knaeps et al. (2015)	427	733	3.5%	0.58 [0.55; 0.62]
Total (95% CI)		**5888**	**24.1%**	**0.54 [0.34; 0.74]**
Heterogeneity: $Tau^2 = 0.0769$; $Chi^2 = 1169.98$, df = 6 ($P < 0.01$); $I^2 = 99\%$				
JBI study quality = Medium				
Ali et al. (2011)	89	99	3.3%	0.90 [0.82; 0.95]
Bonsaksen et al. (2016)	48	87	3.3%	0.55 [0.44; 0.66]
Drebing et al. (2004)	120	228	3.4%	0.53 [0.46; 0.59]
Hatfield et al. (1992)	29	59	3.2%	0.49 [0.36; 0.63]
Henry et al. (2006)	145	374	3.4%	0.39 [0.34; 0.44]
Hölzle et al. (2018)	63	82	3.3%	0.77 [0.66; 0.85]
Iyer et al. (2011)	52	68	3.2%	0.76 [0.65; 0.86]
Khare et al. (2020)	164	212	3.4%	0.77 [0.71; 0.83]
Laudet et al. (2002)	62	130	3.3%	0.48 [0.39; 0.57]
Macias et al. (2001)	117	166	3.4%	0.70 [0.63; 0.77]
McQuilken et al. (2003)	170	310	3.4%	0.55 [0.49; 0.60]
Ramsay et al. (2011)	80	100	3.3%	0.80 [0.71; 0.87]
Rennhack et al. (2021)	40	98	3.3%	0.41 [0.31; 0.51]
Secker et al. (2001)	89	149	3.4%	0.60 [0.51; 0.68]
Secker, & Gelling (2006)	137	193	3.4%	0.71 [0.64; 0.77]
Serowik et al. (2014)	20	49	3.1%	0.41 [0.27; 0.56]
Westcott et al. (2015)	132	167	3.4%	0.79 [0.72; 0.85]
Zaniboni et al. (2011)	39	130	3.3%	0.30 [0.22; 0.39]
Total (95% CI)		**2701**	**59.9%**	**0.61 [0.53; 0.70]**
Heterogeneity: $Tau^2 = 0.0321$; $Chi^2 = 323.18$, df = 17 ($P < 0.01$); $I^2 = 95\%$				
JBI study quality = Low				
Camardese & Youngman (1996)	44	100	3.3%	0.44 [0.34; 0.54]
Filia et al. (2021)	14	16	2.5%	0.88 [0.62; 0.98]
Graffam & Naccarella (1997)	62	91	3.3%	0.68 [0.58; 0.78]
Mueser et al. (2001)	137	233	3.4%	0.59 [0.52; 0.65]
Poremski et al. (2015)	1539	2000	3.5%	0.77 [0.75; 0.79]
Total (95% CI)		**2440**	**16.0%**	**0.66 [0.52; 0.79]**
Heterogeneity: $Tau^2 = 0.0231$; $Chi^2 = 75.48$, df = 4 ($P < 0.01$); $I^2 = 95\%$				
**Total (95% CI)**		**11029**	**100.0%**	**0.61 [0.53; 0.68]**
**Prediction interval**				**[0.21; 0.94]**
Heterogeneity: $Tau^2 = 0.0401$; $Chi^2 = 2093.39$, df = 29 ($P = 0$); $I^2 = 99\%$				
Test for subgroup differences: $Chi^2 = 0.93$, df = 2 ($P = 0.63$)				

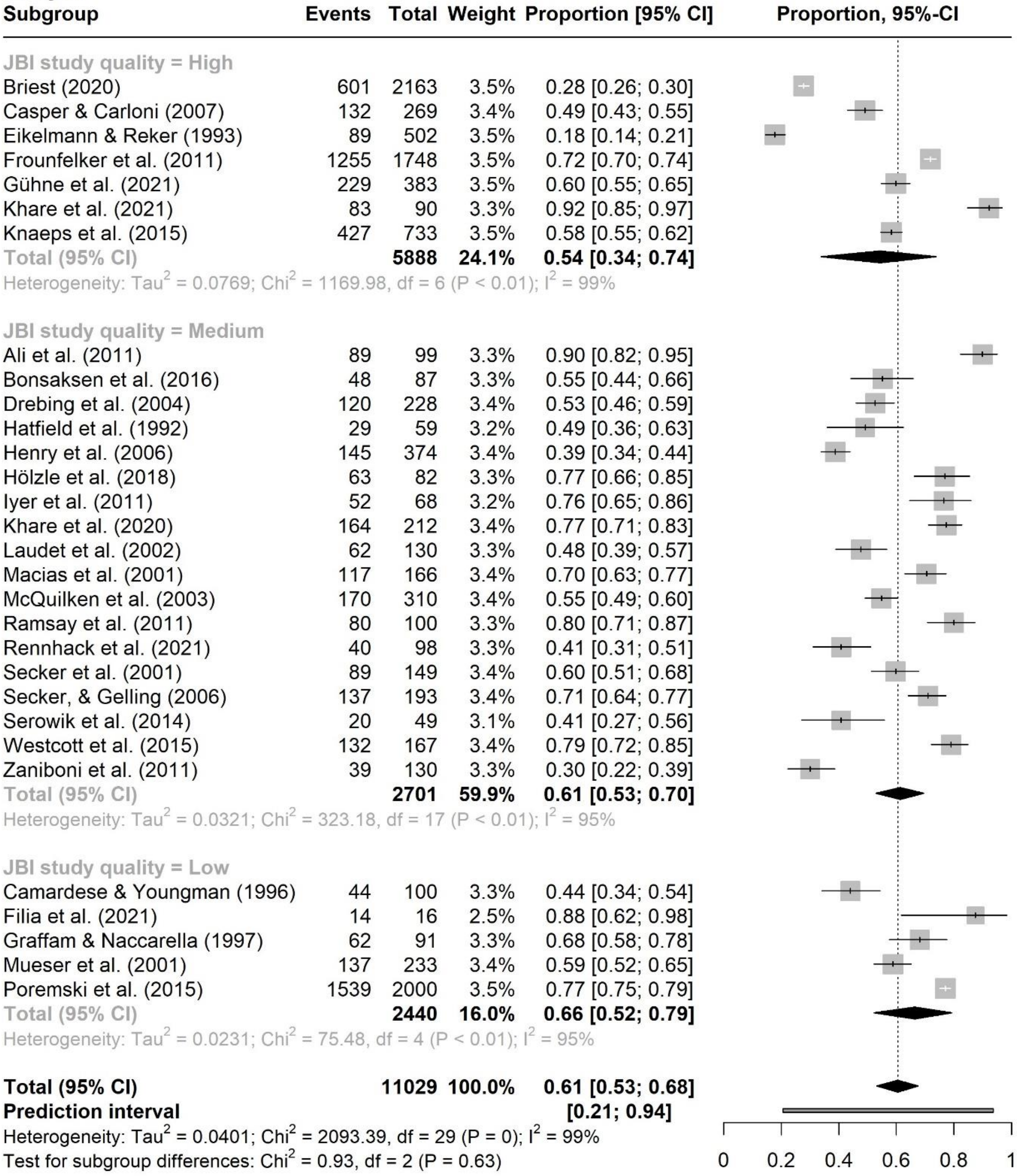

Fig. S2 Subgroup analysis of support settings

Study or Subgroup	Events	Total	Weight	Proportion [95% CI]
**Support setting = Vocational rehabilitation settings**				
Drebing et al. (2004)	120	228	3.4%	0.53 [0.46; 0.59]
Eikelmann & Reker (1993)	89	502	3.5%	0.18 [0.14; 0.21]
Graffam & Naccarella (1997)	62	91	3.3%	0.68 [0.58; 0.78]
Macias et al. (2001)	117	166	3.4%	0.70 [0.63; 0.77]
Rennhack et al. (2021)	40	98	3.3%	0.41 [0.31; 0.51]
Zaniboni et al. (2011)	39	130	3.3%	0.30 [0.22; 0.39]
**Total (95% CI)**		**1215**	**20.2%**	**0.46 [0.29; 0.64]**
Heterogeneity: $Tau^2 = 0.0474$; $Chi^2 = 235.74$, df = 5 ($P < 0.01$); $I^2 = 98\%$				
**Support setting = Community mental health and other settings**				
Ali et al. (2011)	89	99	3.3%	0.90 [0.82; 0.95]
Bonsaksen et al. (2016)	48	87	3.3%	0.55 [0.44; 0.66]
Briest (2020)	601	2163	3.5%	0.28 [0.26; 0.30]
Camardese & Youngman (1996)	44	100	3.3%	0.44 [0.34; 0.54]
Casper & Carloni (2007)	132	269	3.4%	0.49 [0.43; 0.55]
Frounfelker et al. (2011)	1255	1748	3.5%	0.72 [0.70; 0.74]
Hatfield et al. (1992)	29	59	3.2%	0.49 [0.36; 0.63]
Henry et al. (2006)	145	374	3.4%	0.39 [0.34; 0.44]
Laudet et al. (2002)	62	130	3.3%	0.48 [0.39; 0.57]
McQuilken et al. (2003)	170	310	3.4%	0.55 [0.49; 0.60]
Poremski et al. (2015)	1539	2000	3.5%	0.77 [0.75; 0.79]
Secker et al. (2001)	89	149	3.4%	0.60 [0.51; 0.68]
Secker, & Gelling (2006)	137	193	3.4%	0.71 [0.64; 0.77]
Westcott et al. (2015)	132	167	3.4%	0.79 [0.72; 0.85]
**Total (95% CI)**		**7848**	**47.3%**	**0.59 [0.49; 0.68]**
Heterogeneity: $Tau^2 = 0.0339$; $Chi^2 = 1499.77$, df = 13 ($P < 0.01$); $I^2 = 99\%$				
**Support setting = Psychiatric treatment settings**				
Filia et al. (2021)	14	16	2.5%	0.88 [0.62; 0.98]
Gühne et al. (2021)	229	383	3.5%	0.60 [0.55; 0.65]
Hölzle et al. (2018)	63	82	3.3%	0.77 [0.66; 0.85]
Iyer et al. (2011)	52	68	3.2%	0.76 [0.65; 0.86]
Khare et al. (2020)	164	212	3.4%	0.77 [0.71; 0.83]
Khare et al. (2021)	83	90	3.3%	0.92 [0.85; 0.97]
Knaeps et al. (2015)	427	733	3.5%	0.58 [0.55; 0.62]
Mueser et al. (2001)	137	233	3.4%	0.59 [0.52; 0.65]
Ramsay et al. (2011)	80	100	3.3%	0.80 [0.71; 0.87]
Serowik et al. (2014)	20	49	3.1%	0.41 [0.27; 0.56]
**Total (95% CI)**		**1966**	**32.5%**	**0.71 [0.61; 0.80]**
Heterogeneity: $Tau^2 = 0.0257$; $Chi^2 = 116.49$, df = 9 ($P < 0.01$); $I^2 = 92\%$				
**Total (95% CI)**		**11029**	**100.0%**	**0.61 [0.53; 0.68]**
**Prediction interval**				**[0.21; 0.94]**
Heterogeneity: $Tau^2 = 0.0401$; $Chi^2 = 2093.39$, df = 29 ($P = 0$); $I^2 = 99\%$				
Test for subgroup differences: $Chi^2 = 6.75$, df = 2 ($P = 0.03$)				

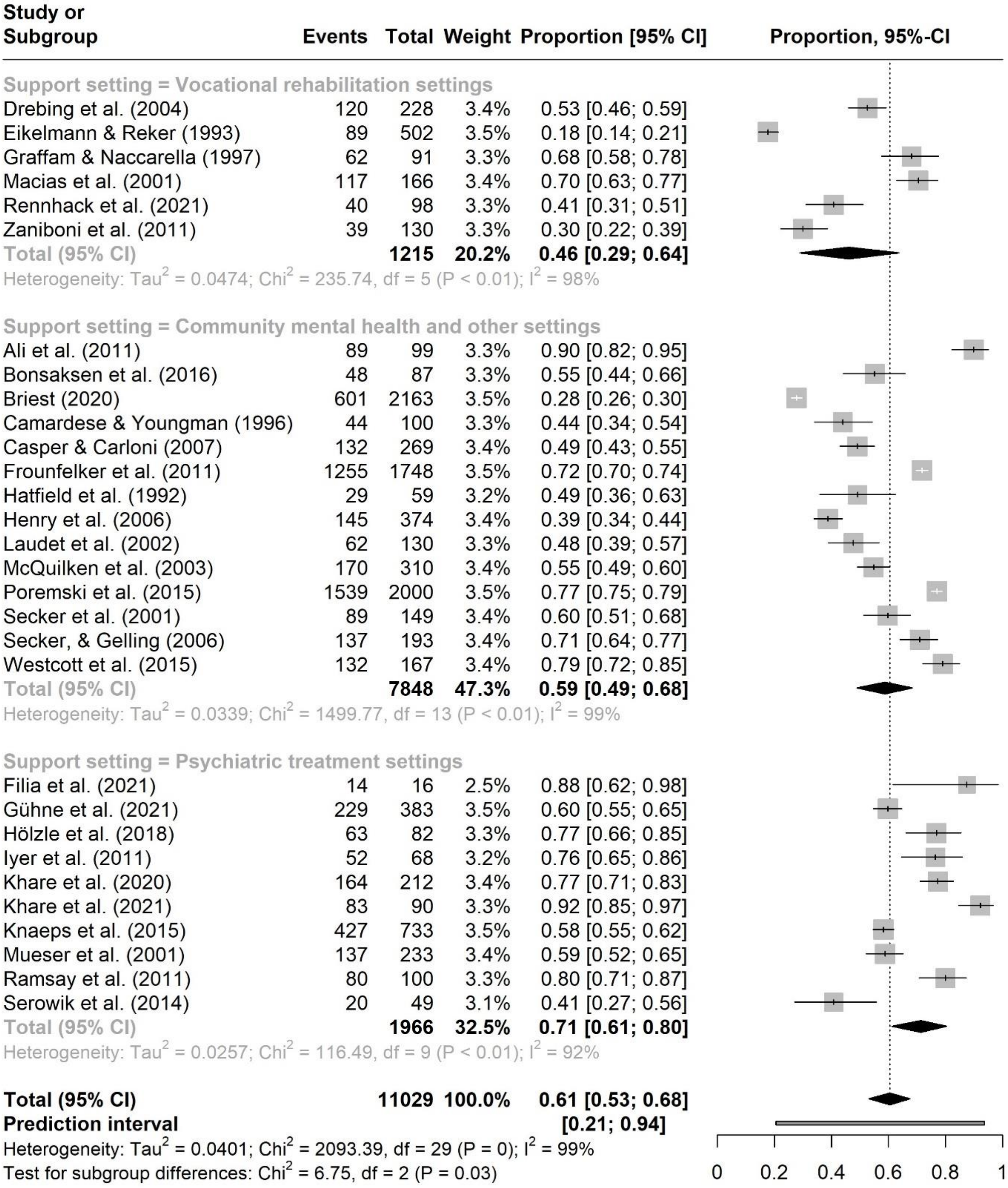

Fig. S3 Subgroup analyses of schizophrenic disorders

Study or Subgroup	Events	Total	Weight	Proportion [95% CI]
**Schizophrenic disorders = Less than 50% of the sample**				
Drebing et al. (2004)	120	228	3.4%	0.53 [0.46; 0.59]
Filia et al. (2021)	14	16	2.5%	0.88 [0.62; 0.98]
Frounfelker et al. (2011)	1255	1748	3.5%	0.72 [0.70; 0.74]
Graffam & Naccarella (1997)	62	91	3.3%	0.68 [0.58; 0.78]
Gühne et al. (2021)	229	383	3.5%	0.60 [0.55; 0.65]
Hölzle et al. (2018)	63	82	3.3%	0.77 [0.66; 0.85]
Knaeps et al. (2015)	427	733	3.5%	0.58 [0.55; 0.62]
Laudet et al. (2002)	62	130	3.3%	0.48 [0.39; 0.57]
Poremski et al. (2015)	1539	2000	3.5%	0.77 [0.75; 0.79]
Rennhack et al. (2021)	40	98	3.3%	0.41 [0.31; 0.51]
Serowik et al. (2014)	20	49	3.1%	0.41 [0.27; 0.56]
Zaniboni et al. (2011)	39	130	3.3%	0.30 [0.22; 0.39]
**Total (95% CI)**		**5688**	**39.5%**	**0.59 [0.49; 0.69]**
Heterogeneity: $Tau^2 = 0.0269$; $Chi^2 = 308.13$, df = 11 ($P < 0.01$); $I^2 = 96\%$				
**Schizophrenic disorders = More than 50% of the sample**				
Eikelmann & Reker (1993)	89	502	3.5%	0.18 [0.14; 0.21]
Hatfield et al. (1992)	29	59	3.2%	0.49 [0.36; 0.63]
Iyer et al. (2011)	52	68	3.2%	0.76 [0.65; 0.86]
Khare et al. (2020)	164	212	3.4%	0.77 [0.71; 0.83]
Khare et al. (2021)	83	90	3.3%	0.92 [0.85; 0.97]
Macias et al. (2001)	117	166	3.4%	0.70 [0.63; 0.77]
McQuilken et al. (2003)	170	310	3.4%	0.55 [0.49; 0.60]
Mueser et al. (2001)	137	233	3.4%	0.59 [0.52; 0.65]
Ramsay et al. (2011)	80	100	3.3%	0.80 [0.71; 0.87]
Westcott et al. (2015)	132	167	3.4%	0.79 [0.72; 0.85]
**Total (95% CI)**		**1907**	**33.5%**	**0.66 [0.52; 0.79]**
Heterogeneity: $Tau^2 = 0.0542$; $Chi^2 = 547.4$, df = 9 ($P < 0.01$); $I^2 = 98\%$				
**Schizophrenic disorders = NA**				
Ali et al. (2011)	89	99	3.3%	0.90 [0.82; 0.95]
Bonsaksen et al. (2016)	48	87	3.3%	0.55 [0.44; 0.66]
Briest (2020)	601	2163	3.5%	0.28 [0.26; 0.30]
Camardese & Youngman (1996)	44	100	3.3%	0.44 [0.34; 0.54]
Casper & Carloni (2007)	132	269	3.4%	0.49 [0.43; 0.55]
Henry et al. (2006)	145	374	3.4%	0.39 [0.34; 0.44]
Secker et al. (2001)	89	149	3.4%	0.60 [0.51; 0.68]
Secker, & Gelling (2006)	137	193	3.4%	0.71 [0.64; 0.77]
**Total (95% CI)**		**3434**	**27.0%**	**0.55 [0.40; 0.69]**
Heterogeneity: $Tau^2 = 0.0433$; $Chi^2 = 375.94$, df = 7 ($P < 0.01$); $I^2 = 98\%$				
**Total (95% CI)**		**11029**	**100.0%**	**0.61 [0.53; 0.68]**
**Prediction interval**				**[0.21; 0.94]**
Heterogeneity: $Tau^2 = 0.0401$; $Chi^2 = 2093.39$, df = 29 ($P = 0$); $I^2 = 99\%$				
Test for subgroup differences: $Chi^2 = 1.26$, df = 2 ($P = 0.53$)				

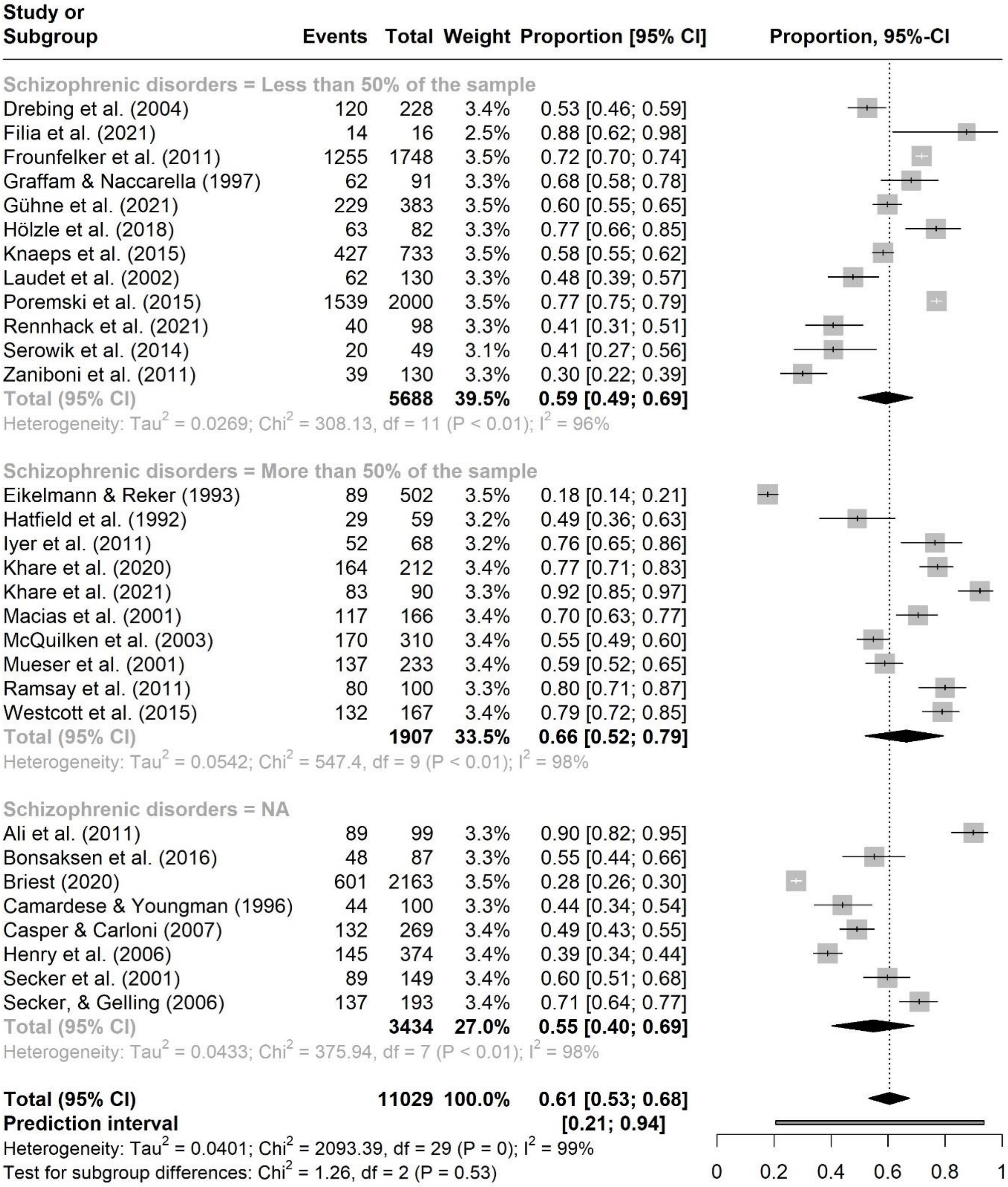

Fig. S4 Subgroup analyses of assessment methods

Study or Subgroup	Events	Total	Weight	Proportion [95% CI]
Assessment method = Closed-ended				
Ali et al. (2011)	89	99	3.3%	0.90 [0.82; 0.95]
Bonsaksen et al. (2016)	48	87	3.3%	0.55 [0.44; 0.66]
Briest (2020)	601	2163	3.5%	0.28 [0.26; 0.30]
Casper & Carloni (2007)	132	269	3.4%	0.49 [0.43; 0.55]
Eikelmann & Reker (1993)	89	502	3.5%	0.18 [0.14; 0.21]
Filia et al. (2021)	14	16	2.5%	0.88 [0.62; 0.98]
Frounfelker et al. (2011)	1255	1748	3.5%	0.72 [0.70; 0.74]
Graffam & Naccarella (1997)	62	91	3.3%	0.68 [0.58; 0.78]
Gühne et al. (2021)	229	383	3.5%	0.60 [0.55; 0.65]
Hatfield et al. (1992)	29	59	3.2%	0.49 [0.36; 0.63]
Henry et al. (2006)	145	374	3.4%	0.39 [0.34; 0.44]
Hölzle et al. (2018)	63	82	3.3%	0.77 [0.66; 0.85]
Khare et al. (2020)	164	212	3.4%	0.77 [0.71; 0.83]
Khare et al. (2021)	83	90	3.3%	0.92 [0.85; 0.97]
Macias et al. (2001)	117	166	3.4%	0.70 [0.63; 0.77]
McQuilken et al. (2003)	170	310	3.4%	0.55 [0.49; 0.60]
Mueser et al. (2001)	137	233	3.4%	0.59 [0.52; 0.65]
Poremski et al. (2015)	1539	2000	3.5%	0.77 [0.75; 0.79]
Ramsay et al. (2011)	80	100	3.3%	0.80 [0.71; 0.87]
Secker, & Gelling (2006)	137	193	3.4%	0.71 [0.64; 0.77]
Total (95% CI)		**9177**	**66.7%**	**0.64 [0.55; 0.73]**
Heterogeneity: $Tau^2 = 0.0460$; $Chi^2 = 1976.09$, df = 19 (P = 0); $I^2 = 99\%$				
Assessment method = Open-ended				
Camardese & Youngman (1996)	44	100	3.3%	0.44 [0.34; 0.54]
Drebing et al. (2004)	120	228	3.4%	0.53 [0.46; 0.59]
Iyer et al. (2011)	52	68	3.2%	0.76 [0.65; 0.86]
Knaeps et al. (2015)	427	733	3.5%	0.58 [0.55; 0.62]
Laudet et al. (2002)	62	130	3.3%	0.48 [0.39; 0.57]
Rennhack et al. (2021)	40	98	3.3%	0.41 [0.31; 0.51]
Secker et al. (2001)	89	149	3.4%	0.60 [0.51; 0.68]
Serowik et al. (2014)	20	49	3.1%	0.41 [0.27; 0.56]
Westcott et al. (2015)	132	167	3.4%	0.79 [0.72; 0.85]
Zaniboni et al. (2011)	39	130	3.3%	0.30 [0.22; 0.39]
Total (95% CI)		**1852**	**33.3%**	**0.53 [0.43; 0.63]**
Heterogeneity: $Tau^2 = 0.0240$; $Chi^2 = 116.23$, df = 9 ($P < 0.01$); $I^2 = 92\%$				
**Total (95% CI)**		**11029**	**100.0%**	**0.61 [0.53; 0.68]**
**Prediction interval**				**[0.21; 0.94]**
Heterogeneity: $Tau^2 = 0.0401$; $Chi^2 = 2093.39$, df = 29 (P = 0); $I^2 = 99\%$				
Test for subgroup differences: $Chi^2 = 2.34$, df = 1 (P = 0.13)				

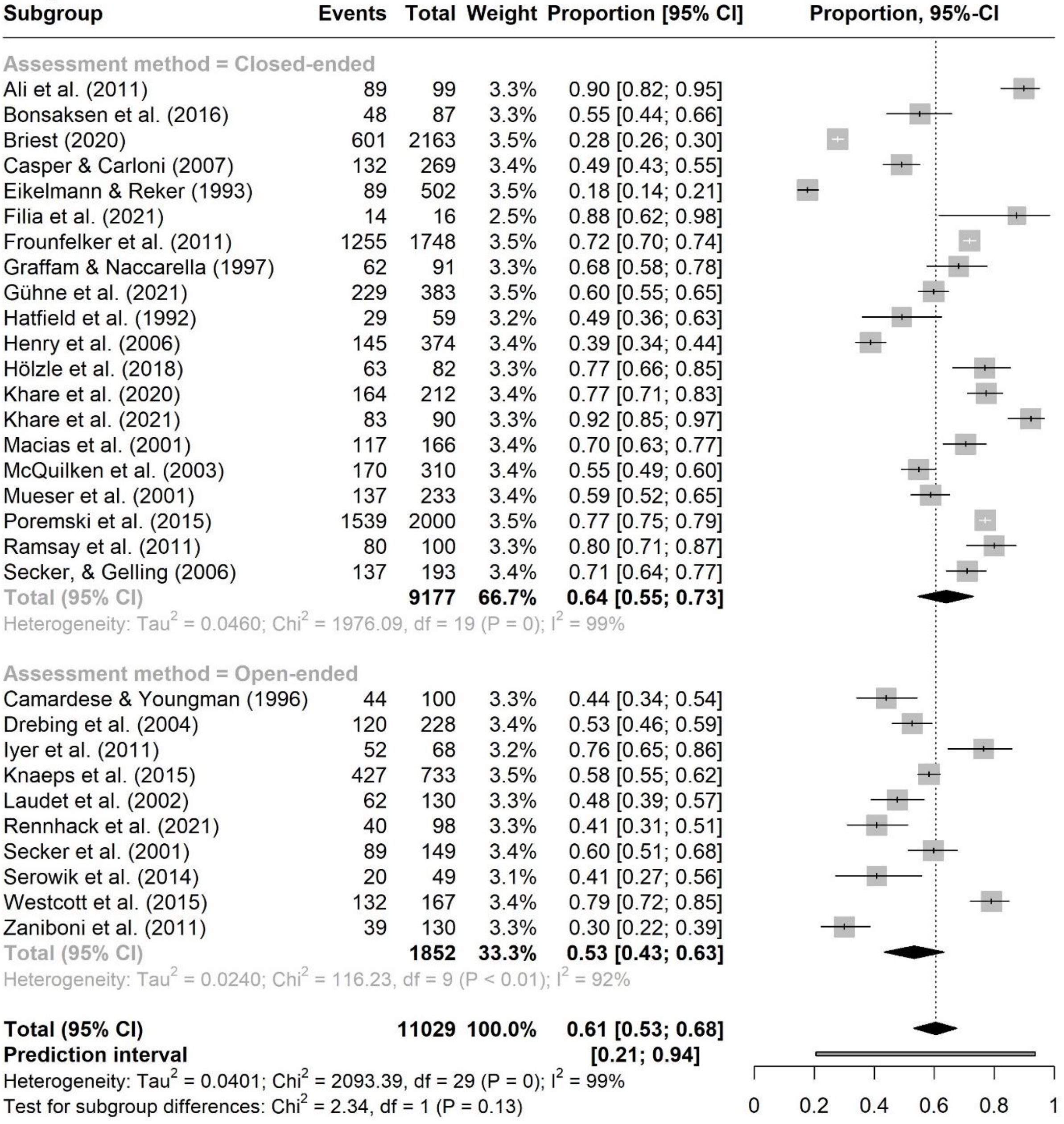

Fig. S5 Subgroup analyses of publication year

Study or Subgroup	Events	Total	Weight	Proportion [95% CI]
Publication year = Before 2008				
Camardese & Youngman (1996)	44	100	3.3%	0.44 [0.34; 0.54]
Casper & Carloni (2007)	132	269	3.4%	0.49 [0.43; 0.55]
Drebing et al. (2004)	120	228	3.4%	0.53 [0.46; 0.59]
Eikelmann & Reker (1993)	89	502	3.5%	0.18 [0.14; 0.21]
Graffam & Naccarella (1997)	62	91	3.3%	0.68 [0.58; 0.78]
Hatfield et al. (1992)	29	59	3.2%	0.49 [0.36; 0.63]
Henry et al. (2006)	145	374	3.4%	0.39 [0.34; 0.44]
Laudet et al. (2002)	62	130	3.3%	0.48 [0.39; 0.57]
Macias et al. (2001)	117	166	3.4%	0.70 [0.63; 0.77]
McQuilken et al. (2003)	170	310	3.4%	0.55 [0.49; 0.60]
Mueser et al. (2001)	137	233	3.4%	0.59 [0.52; 0.65]
Secker et al. (2001)	89	149	3.4%	0.60 [0.51; 0.68]
Secker, & Gelling (2006)	137	193	3.4%	0.71 [0.64; 0.77]
Total (95% CI)		**2804**	**43.9%**	**0.52 [0.44; 0.61]**
Heterogeneity: $Tau^2 = 0.0225$; $Chi^2 = 351.75$, df = 12 (P < 0.01); $I^2 = 97\%$				
Publication year = After 2008				
Ali et al. (2011)	89	99	3.3%	0.90 [0.82; 0.95]
Bonsaksen et al. (2016)	48	87	3.3%	0.55 [0.44; 0.66]
Briest (2020)	601	2163	3.5%	0.28 [0.26; 0.30]
Filia et al. (2021)	14	16	2.5%	0.88 [0.62; 0.98]
Frounfelker et al. (2011)	1255	1748	3.5%	0.72 [0.70; 0.74]
Gühne et al. (2021)	229	383	3.5%	0.60 [0.55; 0.65]
Hölzle et al. (2018)	63	82	3.3%	0.77 [0.66; 0.85]
Iyer et al. (2011)	52	68	3.2%	0.76 [0.65; 0.86]
Khare et al. (2020)	164	212	3.4%	0.77 [0.71; 0.83]
Khare et al. (2021)	83	90	3.3%	0.92 [0.85; 0.97]
Knaeps et al. (2015)	427	733	3.5%	0.58 [0.55; 0.62]
Poremski et al. (2015)	1539	2000	3.5%	0.77 [0.75; 0.79]
Ramsay et al. (2011)	80	100	3.3%	0.80 [0.71; 0.87]
Rennhack et al. (2021)	40	98	3.3%	0.41 [0.31; 0.51]
Serowik et al. (2014)	20	49	3.1%	0.41 [0.27; 0.56]
Westcott et al. (2015)	132	167	3.4%	0.79 [0.72; 0.85]
Zaniboni et al. (2011)	39	130	3.3%	0.30 [0.22; 0.39]
Total (95% CI)		**8225**	**56.1%**	**0.67 [0.56; 0.76]**
Heterogeneity: $Tau^2 = 0.0470$; $Chi^2 = 1605.97$, df = 16 (P = 0); $I^2 = 99\%$				
**Total (95% CI)**		**11029**	**100.0%**	**0.61 [0.53; 0.68]**
**Prediction interval**				**[0.21; 0.94]**
Heterogeneity: $Tau^2 = 0.0401$; $Chi^2 = 2093.39$, df = 29 (P = 0); $I^2 = 99\%$				
Test for subgroup differences: $Chi^2 = 4.57$, df = 1 (P = 0.03)				

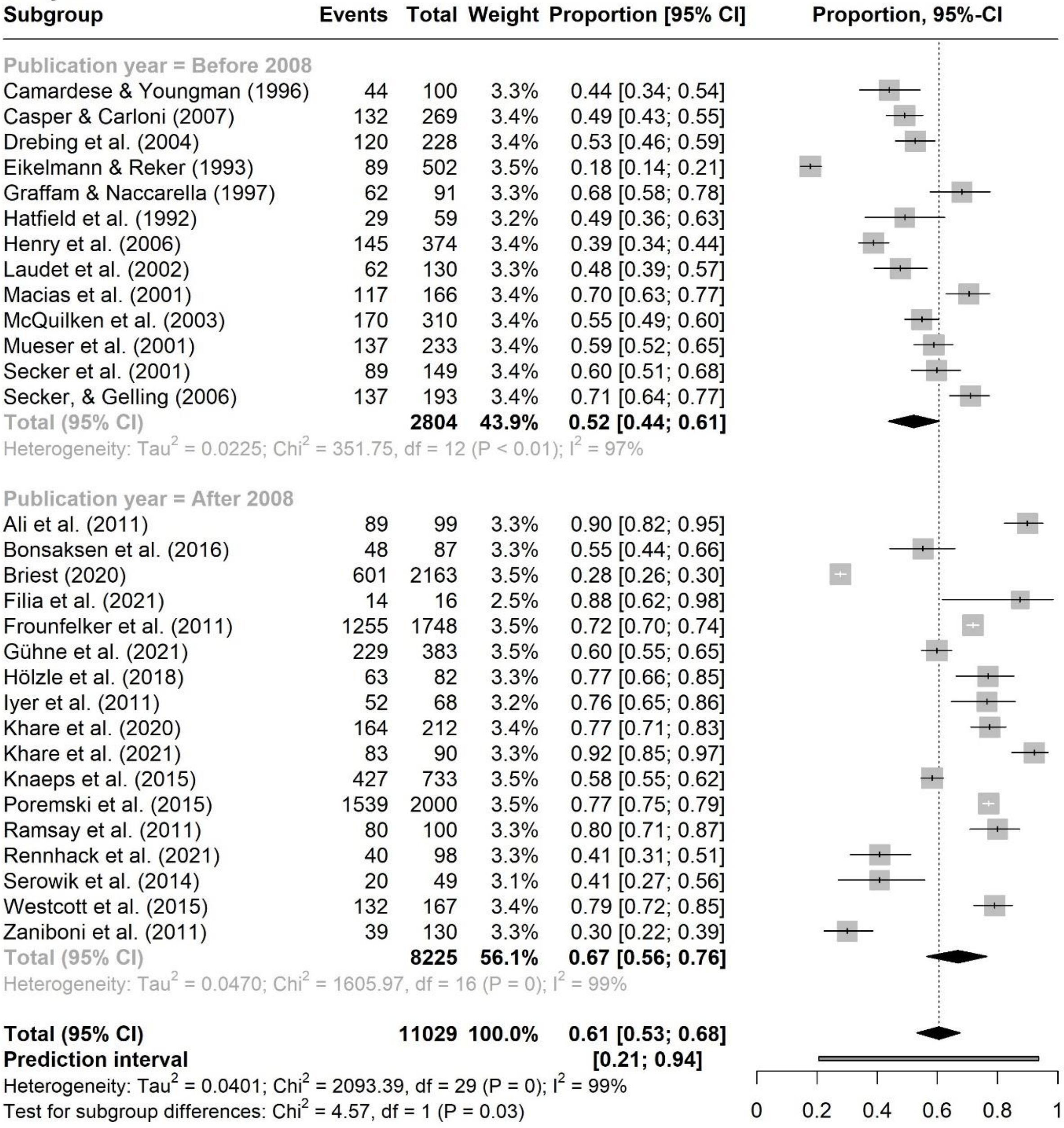

Fig. S6 Subgroup analyses of world regions

Study or Subgroup	Events	Total	Weight	Proportion [95% CI]
World region = America				
Ali et al. (2011)	89	99	3.3%	0.90 [0.82; 0.95]
Camardese & Youngman (1996)	44	100	3.3%	0.44 [0.34; 0.54]
Casper & Carloni (2007)	132	269	3.4%	0.49 [0.43; 0.55]
Drebing et al. (2004)	120	228	3.4%	0.53 [0.46; 0.59]
Frounfelker et al. (2011)	1255	1748	3.5%	0.72 [0.70; 0.74]
Henry et al. (2006)	145	374	3.4%	0.39 [0.34; 0.44]
Laudet et al. (2002)	62	130	3.3%	0.48 [0.39; 0.57]
Macias et al. (2001)	117	166	3.4%	0.70 [0.63; 0.77]
McQuilken et al. (2003)	170	310	3.4%	0.55 [0.49; 0.60]
Mueser et al. (2001)	137	233	3.4%	0.59 [0.52; 0.65]
Poremski et al. (2015)	1539	2000	3.5%	0.77 [0.75; 0.79]
Ramsay et al. (2011)	80	100	3.3%	0.80 [0.71; 0.87]
Serowik et al. (2014)	20	49	3.1%	0.41 [0.27; 0.56]
Total (95% CI)		**5806**	**43.9%**	**0.61 [0.51; 0.70]**
Heterogeneity: $Tau^2 = 0.0290$; $Chi^2 = 417.37$, df = 12 ($P < 0.01$); $I^2 = 97\%$				
World region = Europe				
Bonsaksen et al. (2016)	48	87	3.3%	0.55 [0.44; 0.66]
Briest (2020)	601	2163	3.5%	0.28 [0.26; 0.30]
Eikelmann & Reker (1993)	89	502	3.5%	0.18 [0.14; 0.21]
Gühne et al. (2021)	229	383	3.5%	0.60 [0.55; 0.65]
Hatfield et al. (1992)	29	59	3.2%	0.49 [0.36; 0.63]
Hölzle et al. (2018)	63	82	3.3%	0.77 [0.66; 0.85]
Knaeps et al. (2015)	427	733	3.5%	0.58 [0.55; 0.62]
Rennhack et al. (2021)	40	98	3.3%	0.41 [0.31; 0.51]
Secker et al. (2001)	89	149	3.4%	0.60 [0.51; 0.68]
Secker, & Gelling (2006)	137	193	3.4%	0.71 [0.64; 0.77]
Zaniboni et al. (2011)	39	130	3.3%	0.30 [0.22; 0.39]
Total (95% CI)		**4579**	**37.0%**	**0.49 [0.38; 0.61]**
Heterogeneity: $Tau^2 = 0.0369$; $Chi^2 = 589.63$, df = 10 ($P < 0.01$); $I^2 = 98\%$				
World region = Australia				
Filia et al. (2021)	14	16	2.5%	0.88 [0.62; 0.98]
Graffam & Naccarella (1997)	62	91	3.3%	0.68 [0.58; 0.78]
Westcott et al. (2015)	132	167	3.4%	0.79 [0.72; 0.85]
Total (95% CI)		**274**	**9.2%**	**0.77 [0.67; 0.85]**
Heterogeneity: $Tau^2 = 0.0047$; $Chi^2 = 4.7$, df = 2 ($P = 0.10$); $I^2 = 57\%$				
World region = Asia				
Iyer et al. (2011)	52	68	3.2%	0.76 [0.65; 0.86]
Khare et al. (2020)	164	212	3.4%	0.77 [0.71; 0.83]
Khare et al. (2021)	83	90	3.3%	0.92 [0.85; 0.97]
Total (95% CI)		**370**	**9.9%**	**0.83 [0.71; 0.92]**
Heterogeneity: $Tau^2 = 0.0123$; $Chi^2 = 12.08$, df = 2 ($P < 0.01$); $I^2 = 83\%$				
**Total (95% CI)**		**11029**	**100.0%**	**0.61 [0.53; 0.68]**
**Prediction interval**				**[0.21; 0.94]**
Heterogeneity: $Tau^2 = 0.0401$; $Chi^2 = 2093.39$, df = 29 ($P = 0$); $I^2 = 99\%$				
Test for subgroup differences: $Chi^2 = 20.92$, df = 3 ($P < 0.01$)				

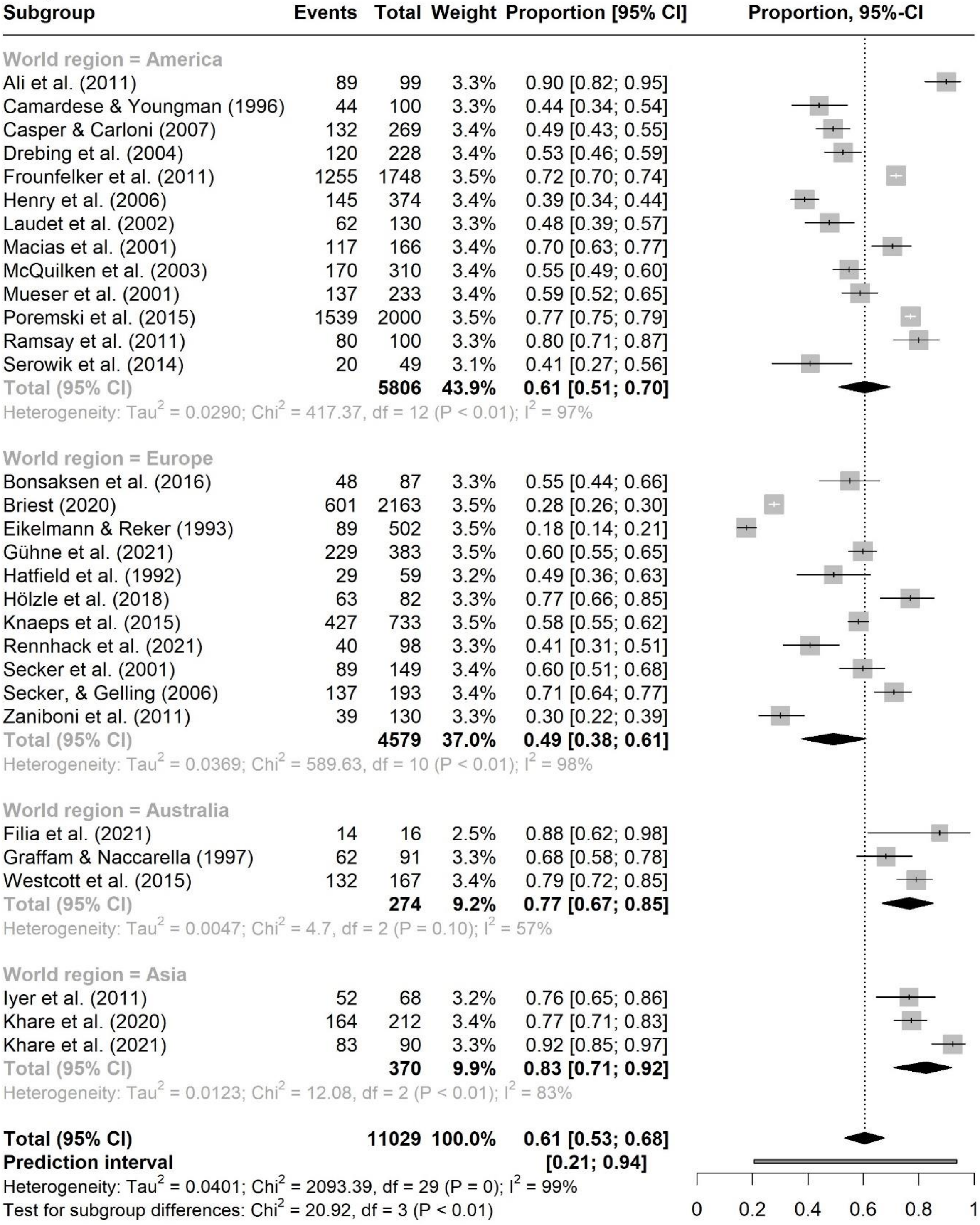

Sensitivity Analyses

Fig. S7 Sensitivity analysis of the pooled proportion of people with mental disorders who prefer competitive employment after exclusion of low quality studies (JBI score of 3 or lower)

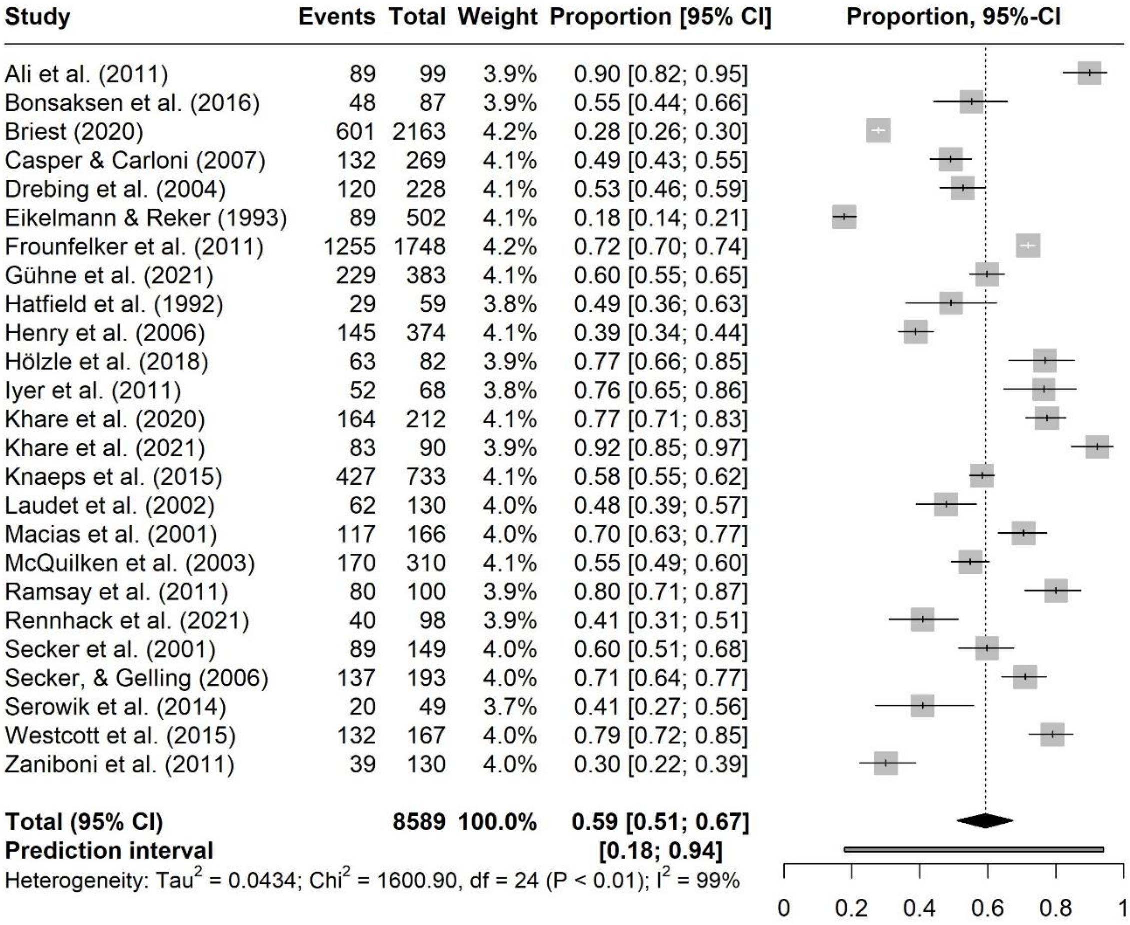

Study	Events	Total	Weight	Proportion [95% CI]
Ali et al. (2011)	89	99	3.9%	0.90 [0.82; 0.95]
Bonsaksen et al. (2016)	48	87	3.9%	0.55 [0.44; 0.66]
Briest (2020)	601	2163	4.2%	0.28 [0.26; 0.30]
Casper & Carloni (2007)	132	269	4.1%	0.49 [0.43; 0.55]
Drebing et al. (2004)	120	228	4.1%	0.53 [0.46; 0.59]
Eikelmann & Reker (1993)	89	502	4.1%	0.18 [0.14; 0.21]
Frounfelker et al. (2011)	1255	1748	4.2%	0.72 [0.70; 0.74]
Gühne et al. (2021)	229	383	4.1%	0.60 [0.55; 0.65]
Hatfield et al. (1992)	29	59	3.8%	0.49 [0.36; 0.63]
Henry et al. (2006)	145	374	4.1%	0.39 [0.34; 0.44]
Hölzle et al. (2018)	63	82	3.9%	0.77 [0.66; 0.85]
Iyer et al. (2011)	52	68	3.8%	0.76 [0.65; 0.86]
Khare et al. (2020)	164	212	4.1%	0.77 [0.71; 0.83]
Khare et al. (2021)	83	90	3.9%	0.92 [0.85; 0.97]
Knaeps et al. (2015)	427	733	4.1%	0.58 [0.55; 0.62]
Laudet et al. (2002)	62	130	4.0%	0.48 [0.39; 0.57]
Macias et al. (2001)	117	166	4.0%	0.70 [0.63; 0.77]
McQuilken et al. (2003)	170	310	4.1%	0.55 [0.49; 0.60]
Ramsay et al. (2011)	80	100	3.9%	0.80 [0.71; 0.87]
Rennhack et al. (2021)	40	98	3.9%	0.41 [0.31; 0.51]
Secker et al. (2001)	89	149	4.0%	0.60 [0.51; 0.68]
Secker, & Gelling (2006)	137	193	4.0%	0.71 [0.64; 0.77]
Serowik et al. (2014)	20	49	3.7%	0.41 [0.27; 0.56]
Westcott et al. (2015)	132	167	4.0%	0.79 [0.72; 0.85]
Zaniboni et al. (2011)	39	130	4.0%	0.30 [0.22; 0.39]
**Total (95% CI)**		**8589**	**100.0%**	**0.59 [0.51; 0.67]**
**Prediction interval**				**[0.18; 0.94]**

Heterogeneity: $Tau^2 = 0.0434$; $Chi^2 = 1600.90$, df = 24 (P < 0.01); $I^2 = 99\%$

Fig. S8 Sensitivity analysis of the pooled proportion of people with mental disorders who prefer competitive employment after exclusion of studies with inadequate recruitment methods (JBI Q2)

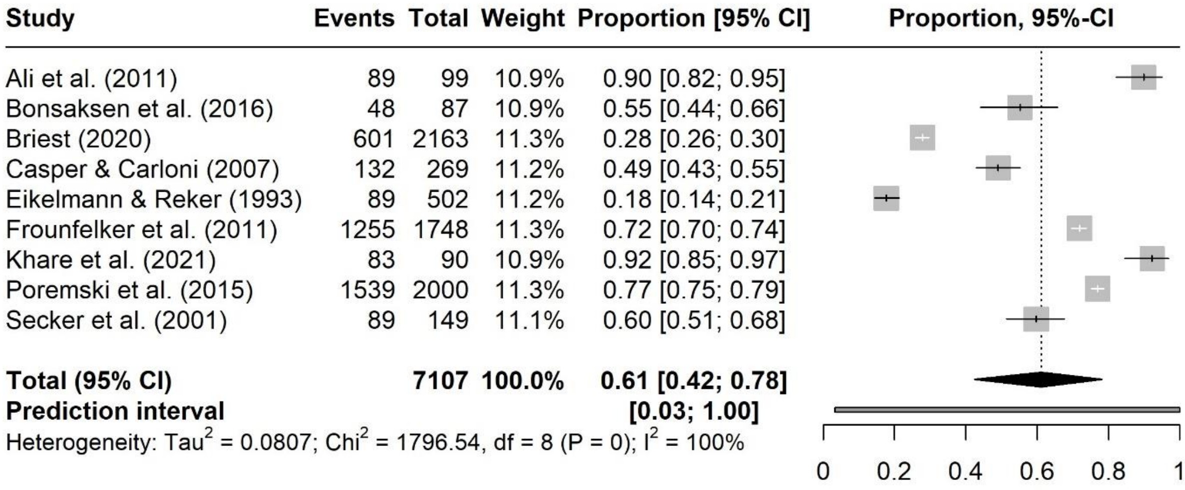

Study	Events	Total	Weight	Proportion [95% CI]
Ali et al. (2011)	89	99	10.9%	0.90 [0.82; 0.95]
Bonsaksen et al. (2016)	48	87	10.9%	0.55 [0.44; 0.66]
Briest (2020)	601	2163	11.3%	0.28 [0.26; 0.30]
Casper & Carloni (2007)	132	269	11.2%	0.49 [0.43; 0.55]
Eikelmann & Reker (1993)	89	502	11.2%	0.18 [0.14; 0.21]
Frounfelker et al. (2011)	1255	1748	11.3%	0.72 [0.70; 0.74]
Khare et al. (2021)	83	90	10.9%	0.92 [0.85; 0.97]
Poremski et al. (2015)	1539	2000	11.3%	0.77 [0.75; 0.79]
Secker et al. (2001)	89	149	11.1%	0.60 [0.51; 0.68]
**Total (95% CI)**		**7107**	**100.0%**	**0.61 [0.42; 0.78]**
**Prediction interval**				**[0.03; 1.00]**

Heterogeneity: $Tau^2 = 0.0807$; $Chi^2 = 1796.54$, df = 8 (P = 0); $I^2 = 100\%$

Excluded papers

Table S5 Citations of the excluded studies, their sources, and the reasons for exclusion

Citation	Source	Reason for Exclusion
Kleinova R. Work aspirations of youth with reduced work ability in the conditions of unemployment. Psychologia a Patopsychologia Dietata 1997; 32(2): 203-10.	database search	A. Article not reached
Han SS, Han JH, Yun EK. [Predictors of employment intention for mentally disabled persons]. Taehan Kanho Hakhoe Chi 2008; 38(4): 541-9.	database search	B. Not written in Latin letters
Asmundsdottir EE. The Worker Role Interview: A powerful tool in Icelandic work rehabilitation. Work: Journal of Prevention, Assessment & Rehabilitation 2004; 22(1): 21-6.	database search	C. Qualitative study
Delman J, Adams LB. Barriers to and facilitators of vocational development for Black young adults with serious mental illnesses. Psychiatric Rehabilitation Journal. 2022;45(1):1-10.	database search	C. Qualitative study
Engdahl P, Svedberg P, Bejerholm U. Acceptability of a digital return-to-work intervention for common mental disorders: A qualitative study on service user perspectives. BMC Psychiatry. 2021;21.	database search	C. Qualitative study
Flinn S, Ventura D, Bonder B. Return to work experiences for veterans with severe mental illness living in rural group home facilities. Work: Journal of Prevention, Assessment & Rehabilitation 2005; 24(1): 63-70.	database search	C. Qualitative study
Holmlund L, Hellman T, Engblom M, Kwak L, Sandman L, Törnkvist L, et al. Coordination of return-to-work for employees on sick leave due to common mental disorders: facilitators and barriers. Disabil Rehabil. 2022;44(13):3113-21.	database search	C. Qualitative study
Joosen MCW, Lugtenberg M, Arends I, van Gestel HJAWM, Schaapveld B, Terluin B, et al. Barriers and facilitators for return to work from the perspective of workers with common mental disorders with short, medium and long-term sickness absence: A longitudinal qualitative study. J Occup Rehabil. 2022;32(2):272-83.	database search	C. Qualitative study
Nagle S, Cook JV, Polatajko HJ. I'm doing as much as I can: Occupational choices of persons with a severe and persistent mental illness. Journal of Occupational Science 2002; 9(2): 72-81.	database search	C. Qualitative study
Anton S. Influence of work attitudes on working Ability among those affected by posttraumatic stress disorder. Journal of Loss and Trauma 2006; 11(4): 311-9.	database search	D. No prevalence of preference for competitive employment reported
Audhoe SS, Nieuwenhuijsen K, Hoving JL, Sluiter JK, Frings-Dresen MHW. Perspectives of unemployed workers with mental health problems: Barriers to and solutions for return to work. Disability & Rehabilitation 2018; 40(1): 28-34.	database search	D. No prevalence of preference for competitive employment reported
Baker AE, Procter NG. Losses related to everyday occupations for adults affected by mental illness. Scand J Occup Ther 2014; 21(4): 287-94.	database search	D. No prevalence of preference for competitive employment reported
Batastini AB, Leuty ME, Davis RM, Jones ACT. Individual and situational factors predicting employment status among revoked community-released offenders. Psychological Services. 2021;18(4):454-63.	database search	D. No prevalence of preference for competitive employment reported

Baumann M, Meyers R, Le Bihan E, Houssemand C. Mental health (GHQ12; CES-D) and attitudes towards the value of work among inmates of a semi-open prison and the long-term unemployed in Luxembourg. BMC Public Health 2008; 8: 214.	database search	D. No prevalence of preference for competitive employment reported
Becker D, Whitley R, Bailey EL, Drake RE. Long-term employment trajectories among participants with severe mental illness in supported employment. Psychiatric Services 2007; 58(7): 922-8.	database search	D. No prevalence of preference for competitive employment reported
Becker DR, Bebout RR, Drake RE. Job preferences of people with severe mental illness: A replication. Psychiatric Rehabilitation Journal 1998; 22(1): 46-50.	database search	D. No prevalence of preference for competitive employment reported
Becker DR, Drake RE, Farabaugh A, Bond GR. Job preferences of clients with severe psychiatric disorders participating in supported employment programs. Psychiatric Services 1996; 47(11): 1223-6.	database search	D. No prevalence of preference for competitive employment reported
Bedell JR, Draving D, Parrish A, Gervey R, Guastadisegni P. A description and comparison of experiences of people with mental disorders in supported employment and paid prevocational training. Psychiatric Rehabilitation Journal 1998; 21(3): 279-83.	database search	D. No prevalence of preference for competitive employment reported
Bond GR, Campbell K, Becker DR. A test of the occupational matching hypothesis for rehabilitation clients with severe mental illness. Journal of Occupational Rehabilitation 2013; 23(2): 261-9.	database search	D. No prevalence of preference for competitive employment reported
Bond GR, Dietzen LL, McGrew JH, Miller LD. Accelerating entry into supported employment for persons with severe psychiatric disabilities. Rehabilitation Psychology 1995; 40(2): 75-94.	database search	D. No prevalence of preference for competitive employment reported
Borger C, Marrow J, Drake RE, Taylor J. Characteristics of enrollees in the supported employment demonstration. Psychiatr Serv. 2021;72(12):1400-6.	database search	D. No prevalence of preference for competitive employment reported
Braitman A, Counts P, Davenport R, et al. Comparison of barriers to employment for unemployed and employed clients in a case management program: An exploratory study. Psychiatric Rehabilitation Journal 1995; 19(1): 3-8.	database search	D. No prevalence of preference for competitive employment reported
Brantschen E, Kawohl W, Rössler W, Bärtsch B, Nordt C. Supported Employment – Improving competitive employment for people with mental illness: The role of motivation and social network. Journal of Vocational Rehabilitation 2014; 40(1): 41-7.	database search	D. No prevalence of preference for competitive employment reported
Brantschen E, Landolt K, Kawohl W, Rossler W, Bartsch B, Nordt C. Two types of expectancies concerning competitive employment among people with mental illness in supported employment. Journal of Vocational Rehabilitation 2017; 46(2): 195-202.	database search	D. No prevalence of preference for competitive employment reported
Brouwer S, Reneman MF, Bultmann U, van der Klink JJ, Groothoff JW. A prospective study of return to work across health conditions: Perceived work attitude, self-efficacy and perceived social support. Journal of Occupational Rehabilitation 2010; 20(1): 104-12.	database search	D. No prevalence of preference for competitive employment reported
Chen C-M, Wang J-Y, Yeh Y-C, Yang S-Y. Factors affecting employability of patients with schizophrenia who had first participated in vocational training: A pilot study. Psychiatric Quarterly. 2023;94(2):165-78.	database search	D. No prevalence of preference for competitive employment reported

Claudi Jensen AG. Towards a parsimonious program theory of return to work intervention. Work 2013; 44(2): 155-64.	database search	D. No prevalence of preference for competitive employment reported
Creed PA, Hicks RE, Machin MA. Behavioural plasticity and mental health outcomes for long-term unemployed attending occupational training programmes. Journal of Occupational & Organizational Psychology 1998; 71(2): 171-91.	database search	D. No prevalence of preference for competitive employment reported
Davis LL, Resnick SG, Maieritsch KP, et al. Employment outcomes from VA vocational services involving transitional work for veterans with a diagnosis of posttraumatic stress disorder. Psychiatric Rehabilitation Journal 2019; 42(3): 257-67.	database search	D. No prevalence of preference for competitive employment reported
Delman J, Adams LB. Barriers to and facilitators of vocational development for Black young adults with serious mental illnesses. Psychiatric Rehabilitation Journal 2021: No Pagination Specified.	database search	D. No prevalence of preference for competitive employment reported
Diamond H. Vocational decision making in a psychiatric outpatient program. Occupational Therapy in Mental Health 1999; 14(3): 67-80.	database search	D. No prevalence of preference for competitive employment reported
Dixon L, Hoch JS, Clark R, et al. Cost-effectiveness of two vocational rehabilitation programs for persons with severe mental illness. Psychiatric Services 2002; 53(9): 1118-24.	database search	D. No prevalence of preference for competitive employment reported
Dorio J, Guitar A, Solheim L, Dvorkin C, Marine S. Differences in job retention in a supported employment program Chinook clubhouse. Psychiatric Rehabilitation Journal 2002; 25(3): 289-98.	database search	D. No prevalence of preference for competitive employment reported
Drummond A, Coole C, Nouri F, Ablewhite J, Smyth G. Using occupational therapists in vocational clinics in primary care: A feasibility study. BMC Family Practice 2020; 21(1): 1-10.	database search	D. No prevalence of preference for competitive employment reported
Duvdevany I, Rimmerman A. Individuals with work-related disabilities: Locus of control, attitudes toward work, and cooperation with the rehabilitation worker. Journal of Applied Rehabilitation Counseling 1996; 27(2): 30-5.	database search	D. No prevalence of preference for competitive employment reported
Ekberg K, Wahlin C, Persson J, Bernfort L, Oberg B. Is mobility in the labor market a solution to sustainable return to work for some sick listed persons? Journal of Occupational Rehabilitation 2011; 21(3): 355-65.	database search	D. No prevalence of preference for competitive employment reported
Eklund M, Backstrom M. Factor structure and construct validity of the Worker Role Self-Assessment (WRS) when used for people with psychiatric disabilities in Sweden. Evaluation & the Health Professions 2016; 39(3): 299-316.	database search	D. No prevalence of preference for competitive employment reported
Eklund M, Sandlund M. Predictors of valued everyday occupations, empowerment and satisfaction in day centres: Implications for services for persons with psychiatric disabilities. Scandinavian Journal of Caring Sciences 2014; 28(3): 582-90.	database search	D. No prevalence of preference for competitive employment reported
Emsley RA, Seedat S, Stein DJ. Posttraumatic stress disorder and occupational disability in South African Security Force members. Journal of Nervous and Mental Disease 2003; 191(4): 237-41.	database search	D. No prevalence of preference for competitive employment reported
Fineran S, Gruber JE. Youth at work: Adolescent employment and sexual harassment. Child Abuse & Neglect 2009; 33(8): 550-9.	database search	D. No prevalence of preference for competitive employment reported
Fyhn T, Overland S, Reme SE. Predictors of employment in people with moderate to severe mental illness participating in a randomized controlled trial of Individual Placement and Support (IPS). Int J Soc Psychiatry. 2021;67(2):150-7.	database search	D. No prevalence of preference for competitive employment reported

Garachana Carpintero ML, Santamaría-Vázquez M. The relationship between volition and independent occupational performance in people with mental disorders. Occupational Therapy in Mental Health 2017; 33(4): 394-406.	database search	D. No prevalence of preference for competitive employment reported
Gregitis S, Glacken J, Julian C, Underwood K. Comparing working role values of employed and unemployed Clubhouse members. Work: Journal of Prevention, Assessment & Rehabilitation 2010; 36(1): 39-46.	database search	D. No prevalence of preference for competitive employment reported
Gutierrez-Garcia RA, Benjet C, Borges G, Mendez Rios E, Medina-Mora ME. Emerging adults not in education, employment or training (NEET): Socio-demographic characteristics, mental health and reasons for being NEET. BMC Public Health 2018; 18(1): 1201.	database search	D. No prevalence of preference for competitive employment reported
Hanna R, Fiedler RC, Dietrich H, Creitemann B, Heuft G. Goal analysis and goal operationalisation: A group intervention for the enhancement of work motivation. PPmP: Psychotherapie Psychosomatik Medizinische Psychologie 2010; 60(8): 316-25.	database search	D. No prevalence of preference for competitive employment reported
Hedlund A, Kristofferzon ML, Boman E, Nilsson A. Are return to work beliefs, psychological well-being and perceived health related to return-to-work intentions among women on long-term sick leave for common mental disorders? A cross-sectional study based on the theory of planned behaviour. BMC Public Health 2021; 21(1): 535.	database search	D. No prevalence of preference for competitive employment reported
Hedlund A, Nilsson A, Boman E, Kristofferzon M-L. Predictors of return to work and psychological well-being among women during/after long-term sick leave due to common mental disorders - a prospective cohort study based on the theory of planned behaviour. Health & Social Care in the Community. 2022:No-Specified.	database search	D. No prevalence of preference for competitive employment reported
Hillborg H, Svensson T, Danermark B. Towards a working life? Experiences in a rehabilitation process for people with psychiatric disabilities. Scand J Occup Ther 2010; 17(2): 149-61.	database search	D. No prevalence of preference for competitive employment reported
Hönig A. [Job placement for people with severe mental illness. The group experience and employment support in a community mental health institution in Buenos Aires]. Vertex 2015; 26(124): 435-40.	database search	D. No prevalence of preference for competitive employment reported
Horn L, Spronken M, Brouwers EPM, de Reuver RSM, Joosen MCW. Identifying return to work self-efficacy trajectories in employees with mental health problems. J Occup Rehabil. 2022;32(1):64-76.	database search	D. No prevalence of preference for competitive employment reported
Jensen AGC. Towards a parsimonious program theory of return to work intervention. Work: Journal of Prevention, Assessment & Rehabilitation 2013; 44(2): 155-64.	database search	D. No prevalence of preference for competitive employment reported
Kang S-Y, Magura S, Blankertz L, Madison E, Spinelli M. Predictors of engagement in vocational counseling for methadone treatment patients. Substance Use & Misuse 2006; 41(8): 1125-38.	database search	D. No prevalence of preference for competitive employment reported
Kilian R, Muller-Stierlin A, Sohner F, et al. Masculinity norms and occupational role orientations in men treated for depression. PLoS ONE 2020; 15(5): e0233764.	database search	D. No prevalence of preference for competitive employment reported
Kin Wong K, Chiu R, Tang B, Mak D, Liu J, Chiu SN. A randomized controlled trial of a supported employment program for persons with long-term mental illness in Hong Kong. Psychiatric Services 2008; 59(1): 84-90.	database search	D. No prevalence of preference for competitive employment reported

Kirsh B. Organizational culture, climate and person-environment fit: Relationships with employment outcomes for mental health consumers. Work: Journal of Prevention, Assessment & Rehabilitation 2000; 14(2): 109-22.	database search	D. No prevalence of preference for competitive employment reported
Kirsh B. Work, workers, and workplaces: a qualitative analysis of narratives of mental health consumers. Journal of Rehabilitation 2000; 66(4): 24-30.	database search	D. No prevalence of preference for competitive employment reported
Krieshok TS, Hastings S, Ebberwein C, Wettersten K, Owen A. Telling a good story: Using narratives in vocational rehabilitation with veterans. The Career Development Quarterly 1999; 47(3): 204-14.	database search	D. No prevalence of preference for competitive employment reported
Kukla M, Strasburger AM, Salyers MP, Rollins AL, Lysaker PH. Psychosocial outcomes of a pilot study of work-tailored cognitive behavioral therapy intervention for adults with serious mental illness. Journal of Clinical Psychology. 2021;77(3):488-95.	database search	D. No prevalence of preference for competitive employment reported
Lancic F, Majski-Cesarec S, Musil V. [School choice and vocational guidance for schoolchildren with chronic diseases and other health problems]. Arh Hig Rada Toksikol 2010; 61(3): 323-32.	database search	D. No prevalence of preference for competitive employment reported
Langle G, Koster M, Mayenberger M, Gunthner A. [Trial employment: A provisional work setting for psychiatric patients]. Psychiatrische Praxis 2000; 27(4): 176-82.	database search	D. No prevalence of preference for competitive employment reported
Larson JE, Barr LK, Kuwabara SA, Boyle MG, Glenn TL. Process and outcome analysis of a supported employment program for people with psychiatric disabilities. American Journal of Psychiatric Rehabilitation 2007; 10(4): 339-53.	database search	D. No prevalence of preference for competitive employment reported
Latimer EA, Lecomte T, Becker DR, et al. Generalisability of the individual placement and support model of supported employment: Results of a Canadian randomised controlled trial. The British Journal of Psychiatry 2006; 189(1): 65-73.	database search	D. No prevalence of preference for competitive employment reported
Lau B, Shiryaeva O, Ruud T, Victor M. What are they returning to? Psychosocial work environment as a predictor of returning to work among employees in treatment for common mental disorders: A prospective observational pre-post study. PLoS ONE 2019; 14(4): e0215354.	database search	D. No prevalence of preference for competitive employment reported
Mares AS, Rosenheck RA. Attitudes towards employment and employment outcomes among homeless veterans with substance abuse and/or psychiatric problems. American Journal of Psychiatric Rehabilitation 2006; 9(3): 145-66.	database search	D. No prevalence of preference for competitive employment reported
McCrohan NM, Mowbray CT, Bybee D, Harris SN. Employment histories and expectations of persons with psychiatric disorders. Rehabilitation Counseling Bulletin 1994; 38(1): 59-71.	database search	D. No prevalence of preference for competitive employment reported
McCrum BW, Burnside LK, Duffy TL. Organising for work: A job clinic for people with mental health needs. Journal of Mental Health 1997; 6(5): 503-13.	database search	D. No prevalence of preference for competitive employment reported
McGilloway S, Donnelly M. Work, rehabilitation and mental health. Journal of Mental Health 2000; 9(2): 199-210.	database search	D. No prevalence of preference for competitive employment reported
McQueen JM, Turner J. Exploring forensic mental health service users' views on work: An interpretative phenomenological analysis. The British Journal of Forensic Practice 2012; 14(3): 168-79.	database search	D. No prevalence of preference for competitive employment reported

Mervis JE, Lysaker PH, Fiszdon JM, et al. Addressing defeatist beliefs in work rehabilitation. Journal of Mental Health 2016; 25(4): 366-71.	database search	D. No prevalence of preference for competitive employment reported
Merz MA, Szymanski EM. Effects of a vocational rehabilitation-based career workshop on commitment to career choice. Rehabilitation Counseling Bulletin 1997; 41(2): 88-104.	database search	D. No prevalence of preference for competitive employment reported
Meschnig A, von Kardorff E, Klaus S. [Return to work from vocational retraining. A long-term analysis of individual trajectories: biografical and structural conditions of success and failure]. Rehabilitation (Stuttg) 2019; 58(3): 153-62.	database search	D. No prevalence of preference for competitive employment reported
Meshberg-Cohen S, Reid-Quinones K, Black AC, Rosen MI. Veterans' attitudes toward work and disability compensation: Associations with substance abuse. Addictive Behaviors 2014; 39(2): 445-8.	database search	D. No prevalence of preference for competitive employment reported
Millward L, Lutte A, Purvis R. Depression and the perpetuation of an incapacitated identity as an inhibitor of return to work. Journal of Psychiatric and Mental Health Nursing 2005; 12(5): 565-73.	database search	D. No prevalence of preference for competitive employment reported
Mowbray CT, Bybee D, Harris SN, McCrohan N. Predictors of work status and future work orientation in people with a psychiatric disability. Psychiatric Rehabilitation Journal 1995; 19(2): 17.	database search	D. No prevalence of preference for competitive employment reported
Mueser KT, Becker DR, Wolfe R. Supported employment, job preferences, job tenure and satisfaction. Journal of Mental Health 2001; 10(4): 411-7.	database search	D. No prevalence of preference for competitive employment reported
O'Day B, Killeen M, Goldberg S. Not just any job: People with psychiatric disabilities build careers. Journal of Vocational Rehabilitation 2006; 25(2): 119-31.	database search	D. No prevalence of preference for competitive employment reported
Pernice R. Employment attitudes and mental health of long-term unemployed people with disabilities: Implications for rehabilitation counselors. Journal of Applied Rehabilitation Counseling 1997; 28(2): 21-5.	database search	D. No prevalence of preference for competitive employment reported
Reker T, Eikelmann B. [Predictors of success in supported employment programmes--results of a prospective study]. Psychiatrische Praxis 1999; 26(5): 218-23.	database search	D. No prevalence of preference for competitive employment reported
Resnick SG, Neale MS, Rosenheck RA. Impact of public support payments, intensive psychiatric community care, and program fidelity on employment outcomes for people with severe mental illness. Journal of Nervous and Mental Disease 2003; 191(3): 139-44.	database search	D. No prevalence of preference for competitive employment reported
Roberts MM, Pratt CW. A construct validity study of employment readiness in persons with severe mental illness. American Journal of Psychiatric Rehabilitation 2010; 13(1): 40-54.	database search	D. No prevalence of preference for competitive employment reported
Schindler VP. A client-centred, occupation-based occupational therapy programme for adults with psychiatric diagnoses. Occupational Therapy International 2010; 17(3): 105-12.	database search	D. No prevalence of preference for competitive employment reported
Secker J, Membrey H. Promoting mental health through employment and developing healthy workplaces: The potential of natural supports at work. Health Education Research 2003; 18(2): 207-15.	database search	D. No prevalence of preference for competitive employment reported
Sripada RK, Henry J, Yosef M, et al. Occupational functioning and employment services use among VA primary care patients	database search	D. No prevalence of preference for competitive employment reported

with posttraumatic stress disorder. Psychological Trauma: Theory, Research, Practice & Policy 2018; 10(2): 140-3.		
Suzuki Y, Kikuchi E, Watanabe S. Assessment of vocational opportunities and continuing job placement for persons with mental disabilities: Factors indicating levels of necessary support. Work: Journal of Prevention, Assessment & Rehabilitation 2008; 30(2): 185-94.	database search	D. No prevalence of preference for competitive employment reported
Tan P, Hawkins WE, Thomas L. Job satisfaction and intent to continue working among individuals with serious mental illness. Psychological Reports 1999; 85(3): 801-7.	database search	D. No prevalence of preference for competitive employment reported
Taskila T, Steadman K, Gulliford J, Thomas R, Elston R, Bevan S. Working with schizophrenia: Experts' views on barriers and pathways to employment and job retention. Journal of Vocational Rehabilitation 2014; 41(1): 29-44.	database search	D. No prevalence of preference for competitive employment reported
Turner N, Ferguson L, Hill M, et al. Perspectives on paid work among men and women with schizophrenia: The results of a two study comparison in Ireland. Work 2020; 67(2): 507-15.	database search	D. No prevalence of preference for competitive employment reported
Turner N, Lydon C. Psychosocial programming in Ireland based on the Model of Human Occupation: a program evaluation study. Occupational Therapy in Health Care 2008; 22(2/3): 105-14.	database search	D. No prevalence of preference for competitive employment reported
Van Dongen CJ. Quality of life and self-esteem in working and nonworking persons with mental illness. Community Mental Health Journal 1996; 32(6): 535-48.	database search	D. No prevalence of preference for competitive employment reported
van Hoffen MF, Joling CI, Heymans MW, Twisk JW, Roelen CA. Mental health symptoms identify workers at risk of long-term sickness absence due to mental disorders: Prospective cohort study with 2-year follow-up. BMC Public Health 2015; 15: 1235.	database search	D. No prevalence of preference for competitive employment reported
Waghorn G, Saha S, McGrath JJ. Correlates of competitive versus noncompetitive employment among adults with psychotic disorders. Psychiatric Services 2014; 65(4): 476-82.	database search	D. No prevalence of preference for competitive employment reported
Werbart A, Bergstedt A, Levander S. Love, work, and striving for the self in balance: Anaclitic and introjective patients' experiences of change in psychoanalysis. Frontiers in Psychology 2020; 11: 144.	database search	D. No prevalence of preference for competitive employment reported
Wormgoor M, Indahl A, Andersen E, Egeland J. Effectiveness of briefer coping-focused psychotherapy for common mental complaints on work-participation and mental health: A pragmatic randomized trial with 2-year follow-up. Journal of Occupational Rehabilitation 2020; 30(1): 22-39.	database search	D. No prevalence of preference for competitive employment reported
Wu Y-J, Su W-L, Lin Y-H, Chueh C-M, Su C-Y. A comparison of perceptions among employed and unemployed individuals with psychiatric disabilities regarding factors affecting employment. OTJR: Occupation, Participation and Health 2009; 29(3): 105-15.	database search	D. No prevalence of preference for competitive employment reported
Zschucke E, Hessel A, Lippke S. [Temporary Disability Pension from the Perspective of the Individual: Self-Reported Physical and Mental Health, Medical Rehabilitation, and Return to Work Plans]. Rehabilitation (Stuttg) 2016; 55(4): 223-9.	database search	D. No prevalence of preference for competitive employment reported
Carlson L, Rapp CA. Consumer preference and individualized job search. American Journal of Psychiatric Rehabilitation 2007; 10(2): 123-30.	Google Scholar	D. No prevalence of preference for competitive employment reported

de Waal A, Dixon LB, Humensky JL. Association of participant preferences on work and school participation after a first episode of psychosis. Early Intervention in Psychiatry 2017; 12(1-5): 959-63.	Google Scholar	D. No prevalence of preference for competitive employment reported
Rice CD, Howard L, Leese M, Jarrett M, Thornicroft G. Determinants of wanting to seek full versus part-time paid employment among people with severe mental illness. Journal of Mental Health 2009; 18(5): 424-32.	Google Scholar	D. No prevalence of preference for competitive employment reported
Alverson H, Carpenter E, Drake RE. An ethnographic study of job seeking among people with severe mental illness. Psychiatric Rehabilitation Journal 2006; 30(1): 15-22.	reference list	D. No prevalence of preference for competitive employment reported
Anthony WA, Rogers ES, Cohen M, Davies RR. Relationships between psychiatric symptomatology, work skills, and future vocational performance. Psychiatric Services 1995; 46(4): 353-8.	reference list	D. No prevalence of preference for competitive employment reported
Burns T, Catty J, White S, et al. The impact of supported employment and working on clinical and social functioning: results of an international study of individual placement and support. Schizophrenia Bulletin 2009; 35(5): 949-58.	reference list	D. No prevalence of preference for competitive employment reported
Campbell K, Bond GR, Drake RE. Who benefits from supported employment: a meta-analytic study. Schizophrenia Bulletin 2011; 37(2): 370-80.	reference list	D. No prevalence of preference for competitive employment reported
Choi K-H, Fiszdon JM, Bell MD. Beyond cognition: a longitudinal investigation of the role of motivation during a vocational rehabilitation program. The Journal of Nervous and Mental Disease 2013; 201(3): 173-8.	reference list	D. No prevalence of preference for competitive employment reported
Curtin RB, Oberley ET, Sacksteder P, Friedman A. Differences between employed and nonemployed dialysis patients. American Journal of Kidney Diseases 1996; 27(4): 533-40.	reference list	D. No prevalence of preference for competitive employment reported
Grove B. Mental health and employment: shaping a new agenda. Journal of Mental Health 1999; 8(2): 131-40.	reference list	D. No prevalence of preference for competitive employment reported
Honkonen T, Stengård E, Virtanen M, Salokangas RKR. Employment predictors for discharged schizophrenia patients. Social Psychiatry and Psychiatric Epidemiology 2007; 42: 372-80.	reference list	D. No prevalence of preference for competitive employment reported
LaPlante MP, Kennedy J, Kaye HS, Wenger BL. Disability and employment. disability statistics abstract number 11. National Institute on Disability and Rehabilitation Research 1996.	reference list	D. No prevalence of preference for competitive employment reported
McManus S, Bebbington PE, Jenkins R, Brugha T. Mental health and wellbeing in England: the adult psychiatric morbidity survey 2014: NHS digital; 2016.	reference list	D. No prevalence of preference for competitive employment reported
Mueser KT, Clark RE, Haines M, et al. The hartford study of supported employment for persons with severe mental illness: employment and non-vocational outcomes. Journal of Consulting and Clinical Psychology 2004; 72(3): 479-90.	reference list	D. No prevalence of preference for competitive employment reported
Provencher HL, Gregg R, Mead S, Mueser KT. The role of work in the recovery of persons with psychiatric disabilities. Psychiatric Rehabilitation Journal 2002; 26(2): 132-44.	reference list	D. No prevalence of preference for competitive employment reported
Reid AM, Lang CM, O'Neill T. Services for schizophrenic patients and their families: what they say they need. Behavioural and Cognitive Psychotherapy 1993; 21(2): 107-13.	reference list	D. No prevalence of preference for competitive employment reported
Rogers ES, Anthony WA, Toole J, Brown MA. Vocational outcomes following psychosocial rehabilitation: a longitudinal	reference list	D. No prevalence of preference for competitive employment reported

study of three programs. Journal of Vocational Rehabilitation 1991; 1(3): 21-9.		
Saperstein AM, Fiszdon JM, Bell MD. Intrinsic motivation as a predictor of work outcome after vocational rehabilitation in schizophrenia. The Journal of Nervous and Mental Disease 2011; 199(9): 672-7.	reference list	D. No prevalence of preference for competitive employment reported
Waghorn G, Chant D, White P, Whiteford H. Delineating disability, labour force participation and employment restrictions among persons with psychosis. Acta Psychiatrica Scandinavica 2004; 109(4): 279-88.	reference list	D. No prevalence of preference for competitive employment reported
Waghorn G, Chant D, Whiteford H. Clinical and non-clinical predictors of vocational recovery for Australians with psychotic disorders. Journal of Rehabilitation 2002; 68(4): 40-51.	reference list	D. No prevalence of preference for competitive employment reported
Waghorn G, Chant D, Whiteford H. The strength of self-reported course of illness in predicting vocational recovery for persons with schizophrenia. Journal of Vocational Rehabilitation 2003; 18(1): 33-41.	reference list	D. No prevalence of preference for competitive employment reported
Waghorn G, Saha S, Harvey C, et al. 'Earning and learning'in those with psychotic disorders: the second Australian national survey of psychosis. Australian & New Zealand Journal of Psychiatry 2012; 46(8): 774-85.	reference list	D. No prevalence of preference for competitive employment reported
Waghorn GR, Chant DC. Employment restrictions among persons with ICD-10 anxiety disorders: characteristics from a population survey. Journal of Anxiety Disorders 2005; 19(6): 642-57.	reference list	D. No prevalence of preference for competitive employment reported
Watkins KE, Pincus HA, Paddock S, et al. Care for veterans with mental and substance use disorders: good performance, but room to improve on many measures. Health Affairs 2011; 30(11): 2194-203.	reference list	D. No prevalence of preference for competitive employment reported
Zivin K, Bohnert AS, Mezuk B, Ilgen MA, Welsh D, Ratliff S, et al. Employment status of patients in the VA health system: implications for mental health services. Psychiatr Serv. 2011;62(1):35-8.	reference list	D. No prevalence of preference for competitive employment reported
Benz MR, McAllister M. Occupational and leisure preferences of older adults with mental retardation. Australia & New Zealand Journal of Developmental Disabilities 1990; 16(3): 233-44.	database search	E. No (sub-)sample with MD
Creed P. Mental health, attitude-to-work and life situation outcomes for long-term unemployed attending occupational skills training. Journal of Applied Social Behaviour 1997; 4(1): 52-68.	database search	E. No (sub-)sample with MD
Diminic S, Hielscher E, Harris MG. Caring hours and possible need for employment support among primary carers for adults with mental illness: Results from an Australian household survey. Health & Social Care in the Community 2019; 27(5): e837-e49.	database search	E. No (sub-)sample with MD
Hounshell J, Tomori C, Newlin R, et al. Changes in finances, insurance, employment, and lifestyle among persons diagnosed with hairy cell leukemia. Oncologist 2001; 6(5): 435-40.	database search	E. No (sub-)sample with MD
Kessemeier F, Petermann F, Stockler C, Bassler M, Pfeiffer W, Kobelt A. Using mental contrasting with implementation intentions to promote work-related goals in psychosomatic rehabilitation. Zeitschrift fur Klinische Psychologie und Psychotherapie: Forschung und Praxis 2018; 47(2): 89-100.	database search	E. No (sub-)sample with MD

Kilsby MS, Beyer S. Enhancing self-determination in job matching in supported employment for people with learning disabilities: An intervention study. Journal of Vocational Rehabilitation 2002; 17(2): 125-35.	database search	E. No (sub-)sample with MD
Milleur Y, Desveaux J-B. Getting back to work. Topique: La Psychanalyse Aujourd'hui 2020; 148: 63-77.	database search	E. No (sub-)sample with MD
Suijkerbuijk Y, Nieuwenhuijsen K. Identification of the return-to-work mode in unemployed workers with mental health issues: A focus group study among occupational health professionals. Work (Reading, Mass). 2023;74(3):891-906.	database search	E. No (sub-)sample with MD
Szivos SE. Attitudes to work and their relationship to self esteem and aspirations among young adults with a mild mental handicap. British Journal of Mental Subnormality 1990; 36(71): 108-17.	database search	E. No (sub-)sample with MD
Taylor LC. Work attitudes, employment barriers, and mental health symptoms in a sample of rural welfare recipients. American Journal of Community Psychology 2001; 29(3): 443-63.	database search	E. No (sub-)sample with MD
Wehmeyer ML, Bolding N. Self-determination across living and working environments: A matched-samples study of adults with mental retardation. Mental Retardation 1999; 37(5): 353-63.	database search	E. No (sub-)sample with MD
West MD, Targett P, Steininger G, Anglin N. Project Corporate Support (CORPS): A model demonstration project on workplace supports. Journal of Vocational Rehabilitation 2001; 16(2): 111-8.	database search	E. No (sub-)sample with MD
Worsdell AS, Iwata BA, Wallace MD. Duration-based measures of preference for vocational tasks. Journal of Applied Behavior Analysis 2002; 35(3): 287-90.	database search	E. No (sub-)sample with MD
Pernice R, Long N. Long-term unemployment, employment attitudes and mental health. Australian Journal of Social Issues 1996; 31(3): 311-26.	Google Scholar	E. No (sub-)sample with MD
Catty J, Lissouba P, White S, et al. Predictors of employment for people with severe mental illness: results of an international six-centre randomised controlled trial. The British Journal of Psychiatry 2008; 192(3): 224-31.	reference list	E. No (sub-)sample with MD
Khare C, McGurk SR, Fulford D, et al. A longitudinal analysis of employment in people with severe mental illnesses in India. Schizophr Res 2021; 228: 472-80.	database search	F. Duplicate publication of an included study
Poremski D, Hwang SW. Willingness of Housing First participants to consider supported-employment services. Psychiatric Services 2016; 67(6): 667-70.	database search	F. Duplicate publication of an included study
Reker T, Eikelmann B. [The course of illness and rehabilitation of schizophrenic patients in ambulatory vocational therapy. A prospective study over 3 years]. Nervenarzt 1998; 69(3): 210-8.	database search	F. Duplicate publication of an included study
Reker T, Eikelmann B. Work therapy for schizophrenic patients: Results of a 3-year prospective study in Germany. Eur Arch Psychiatry Clin Neurosci 1997; 247(6): 314-9.	database search	F. Duplicate publication of an included study
Reker T, Hornung WP, Schonauer K, Eikelmann B. Long-term psychiatric patients in vocational rehabilitation programmes: A naturalistic follow-up study over 3 years. Acta Psychiatrica Scandinavica 2000; 101(6): 457-63.	database search	F. Duplicate publication of an included study
Wan Kasim SH, Midin M, Abu Bakar AK, Sidi H, Jaafar NRN, Das S. Employment program for patients with severe mental illness in Malaysia: A 3-month outcome. Comprehensive Psychiatry 2014; 55(1): S38-S45.	database search	F. Duplicate publication of an included study
